# Primary structures of different isoforms of buffalo pregnancy-associated glycoproteins (BuPAGs) during early pregnancy and elucidation of the 3-dimensional structure of the most abundant isoform BuPAG 7

**DOI:** 10.1371/journal.pone.0206143

**Published:** 2018-11-07

**Authors:** Masoud Lotfan, Suman Choudhary, Munna Lal Yadav, Sudarshan Kumar, Surender Singh, Shveta Bathla, Preeti Rawat, Jai K. Kaushik, Ashok K. Mohanty

**Affiliations:** Animal Biotechnology Centre, National Dairy Research Institute, Karnal, Haryana, India; International Centre for Genetic Engineering and Biotechnology, ITALY

## Abstract

Pregnancy-associated glycoproteins (PAGs) are expressed during pregnancy by the trophoectodermal cells of fetus. Presence of PAGs in dam's circulation has been widely used in pregnancy diagnosis. The present study reports the identification and characterization of different PAG isoforms in buffalo during early stages of pregnancy. The *PAG* mRNAs isolated from fetal cotyledons (Pregnancy stages: 45, 75 and 90 days) were successfully cloned in pJET1.2 vector and transformed in *E*. *coli*. A total of 360 random clones were sequenced and correlated with their stages of expression. A total of 12 isoforms namely, BuPAG 1, 2, 4, 6, 7, 8, 9, 13, 15, 16, 18 and one new isoform were identified. BuPAG 7 was found as the most abundant isoform in all three stages followed by BuPAG 18. Further, a large number of variants were found for most of these isoforms. Phylogenetic relationship of identified BuPAGs showed that BuPAG 2 belonged to an ancient group while other members clustered with modern group. Three-dimensional (3D) structure of BuPAG 7 was determined by homology modeling and molecular dynamic (MD) simulations which displayed a typical fold represented by other aspartic proteinase (AP) family members. Molecular docking of Pepstatin inhibitor with BuPAG 7 revealed to interact through various hydrogen bonding and hydrophobic interactions. Various amino acid substitutions were observed in peptide-binding cleft of BuPAG 7. Superimposition of BuPAG 7 with homologous structures revealed the presence of a 35–41 amino acid long insertion (alpha helix connected by two loops) near the N- terminus which seems to be a unique feature of BuPAG 7 in AP family. This is the first report on identification and sequence characterization of PAG isoforms in buffalo with unique finding that these isoforms represent many transcript variants. We also report 3D structure of the most abundant isoform BuPAG 7 for the first time.

## Introduction

Placentation in buffalo is of the synepitheliochorial type where placentome develops because of local interactions between fetal placenta and uterine epithelium. The trophoectodermal cells at placentome are structurally and functionally different, some of which are binucleate while others are mononucleate. Both types of cells are involved in synthesis and secretion of pregnancy associated glycoproteins (PAGs) which are released into mother’s blood [1–4]. PAGs are a multigene family of proteins belonging to aspartic proteinase (AP) family and they have been supposed to play role in maternal recognition of pregnancy (MRP) and modulation of immunity [5–10]. Early expression of PAG genes has been reported in conceptus and endometrium of pregnant cows which may have a role in regulating mechanisms related to embryo survival [11]. However, experimental data of evidence supporting the functions of PAGs are very minimal.

PAG genes have been cloned and sequenced in many species such as cattle, sheep, goat, pig and white tailed deer [12–18]. Many PAG isoforms and their variants have been detected in various animal species through cDNA screening and extraction from placental tissues [19–22]. In bovine, 22 PAG cDNA transcripts were reported as early as day 18^th^ of pregnancy [18, 23, 24]. In goat, a total of 11 transcripts were reported from placental tissue [25, 26]. Based upon the site of expression, PAGs are generally categorized into two main groups: PAG-I group which is restricted to the trophoblast binucleate cells and PAG-II group which are secreted by both mono- and binucleate cells of the trophoblast [4, 15, 24, 27, 28]. Some PAG members affect implantation and trophoblast adhesion while other PAG members are involved in remodeling of the feto-maternal unit during placenta development in various mammalian species [29]. Although the function of PAGs has not been well studied, in pig, they act as luteoprotective ligands during implantation by binding to luteal and uterine gonadotropin receptors [30]. PAGs are secreted early in pregnancy immediately post implantation (during day 19 of fertilization in bovine), reach to maternal circulation and serve as specific markers for pregnancy detection [31]. They are considered as preferred molecules for pregnancy detection because of their additional utility for assessing the viability and early embryonic mortality of fetus. The level of PAGs declines in maternal blood and gradually vanishes during early embryonic mortality, thus serving as a unique biomarker for embryo survival [32]. Also, PAGs are not detected in pseudo pregnancy or luteal cystic conditions wherein progesterone can be falsely detected in the sample as a positive indicator of pregnancy.

PAGs in general, are reported to share approximately 50% sequence identity with members of aspartic proteinase family such as pepsin, chymosin, renin, cathepsin D, and E with several conserved regions [33, 34]. The mammalian aspartic proteinases (APs) are bi-lobed proteins with N-terminal and C-terminal lobes which together form a substrate-binding cleft [35, 36]. Various inhibitors such as pepstatin are well known to bind aspartic proteinases such as pepsin, chymosin, cathepsins D and E [37]. The comparative modeling and amino acid substitution analysis has revealed that PAG family includes both active and inactive aspartic proteinases which retain their binding specificities [38].

Efforts have been made in the past to characterize PAGs in pregnant buffalo and also its application as a biomarker for early detection of pregnancy [39]. But the existence of multiple isoforms of PAG, nearly more than 100 genes in bovine [14] makes the choice ambiguous because it is difficult to predict which isoform appears the earliest and hence, should be considered as a biomarker for early detection of pregnancy [13]. In bovine, it is known that different isoforms are expressed differentially at different time points during pregnancy. The expression of PAG genes was analyzed during different stages of pregnancy in cattle where PAG 1 and PAG 2 genes were found to be expressed during one to four months of pregnancy and thus reported as early markers of pregnancy [23, 40]. However, no information is available on the expression of various PAG isoforms, their time of expression and primary structures in buffalo till date. Lack of information is particularly critical in buffalo because very few studies have been reported on buffalo specific PAGs [39]. Despite the multiplicity of PAG genes, protein level evidence is limited to only a few PAG isoforms (PAG 1 and PAG 2) in cattle and only a few reports are available on the characterization of PAGs at the sequence and structural level [41–43].

Considering the lack of information on buffalo-specific PAGs (BuPAGs) and absence of adequate data, we report here the primary structures of different isoforms of PAGs in buffalo, their time of expression during early stages of pregnancy (45 days to 90 days) and relative abundance of individual transcripts during of the progression of pregnancy. Also in view of the absence of experimentally determined structures of PAGs in any species, we describe here the 3D structure of the most abundant BuPAG isoform (BuPAG 7) using homology modeling and molecular dynamic (MD) simulations approach and its interaction with pepstatin inhibitor for the first time.

## Materials and methods

### Sample collection and total RNA extraction

Gravid uteri from pregnant water buffalo (*Bubalus bubalis)* were collected from local slaughter house around Karnal, India. Animals were not slaughtered specifically for sample collection and hence ethical clearance was not required to collect the samples. Fetuses were retrieved and the age of pregnancy was determined by measuring their crown to rump (C-R) length [44]. Individual cotyledons were collected, washed with DEPC-treated water (0.1%) and stored in RNA later solution (Sigma Chem. Co.) at -20°C till further use. All samples were mainly categorized into three groups i.e. 45 days, 75 days and 90 days of pregnancy. Each group contained samples from 4 different fetuses. Total RNA was extracted from the cotyledonary tissue by using TRIzol reagent (Invitrogen, USA) as per manufacturer's instructions. The possible genomic DNA contamination in prepared RNA samples was removed by using DNA free kit (Ambion, USA). The total RNA extracted was quantified by measuring the ratio of absorbance at 260/280 nm wavelength using the Infinite 200 PRO NanoQuant system (Tecan, Austria). The purity and integrity of prepared RNA samples were further verified by 1.2% agarose gel electrophoresis.

Twenty two PAG isoform sequences of *Bos taurus* were retrieved from GenBank nucleotide database (GenBank: L27833.1, NM_176614, XM_615231, NM_176615.2, NM_176616, NM_176617.2, BC133469.1, NM_176619.2, NM_176620.2) and were aligned using Clustal W (1.82) for primer designing. A total of 6 sets of primers were designed for full length amplification of *BuPAGs* by analyzing the conserved sequences in the upstream and downstream regions of ORF, using Primer3 software hosted at NCBI (S1 Table). For the amplification of most of the *PAG* isoforms, one set of primers i.e. BoPAGF(conserved) and BoPAGRcommon was designed by analyzing the most conserved regions of *PAG* isoforms of *Bos taurus* species. For the amplification of other isoforms i.e. 1, 3, 4 and 5; individual forward primers namely, *BoPAGF 1*, *BoPAGF 3*, *BoPAGF 4* and *BoPAGF 5*, were designed keeping the reverse primer same i.e. BoPAG Rcommon. Both the forward and reverse primers span 5’ UTR and 3’UTR regions, respectively. The full length cDNA synthesis was performed using the Thermoscript RT-PCR kit for first strand cDNA synthesis (Invitrogen) following manufacturer’s protocol. The synthesized cDNA was ligated with pJET1.2/blunt vector (Fermentas, USA) as per the manufacturer’s protocol followed by transformation into chemically competent Top10 cells (Invitrogen, USA). The positive clones containing the recombinant plasmids were screened by PCR amplification using the gene-specific primers and plasmids were custom sequenced by capillary sequencing using the SP6 and T7sequencing primers by different sequencing service providers (SciGenom, India and Eurofins genomics, India Pvt. Ltd.) The sequencing of each recombinant plasmid was done in both forward and reverse direction using T7 forward universal sequencing primer and pJET 1.2 reverse sequencing primers, both being part of the vector. The gene sequences of all identified BuPAGs have been submitted to NCBI with following accession numbers: BuPAG 1 (KX611167), BuPAG 2 variant 1–4 (KX611168- KX611171), BuPAG 4 (MH191348), BuPAG 6 (KX611172), BuPAG 7 variant 1–29 (KX611173- KX611200), BuPAG 8 variant 1–5 (KX611201- KX611205), BuPAG 9 (MH191349), BuPAG 13 (KX611206), BuPAG 15 (KX611207), BuPAG 16 variant 1–6 (KX611208- KX611213), BuPAG 18 variant 1–11 (KX611214- KX611224), Novel BuPAG (KX611225).

### Sequence analysis

The sequences were analyzed using DNASTAR Lasergene software. Forward and Reverse sequence data for each recombinant plasmid was aligned on SeqMan module and any mismatch in the sequence was corrected using the quality signal from the chromatogram. Both the forward and reverse gene specific primers were identified on the corrected sequence and the extra sequences on both the extremes were trimmed off. The ORFs were identified by using the ORF finder tool at NCBI (http://www.ncbi.nlm.nih.gov/projects/gorf/) and translated using the translate tool at ExPASy (http://www.expasy.org). The identified protein sequences were compared with protein sequence data of all *PAG* genes for *Bos taurus* and *Bubalus bubalis* available at NCBI GenBank database using the program pBLAST. The sequence nomenclatures were decided on the basis of maximum similarity of the sequences with *Bos Taurus* and *Bubalus bubalis* available in the database. For all the *BuPAG* sequences matching maximally with a particular reported *BuPAG* isoform, multiple alignments were done using the MegAlign module of DNASTAR software to check whether they are the same sequences or they are the variants of a particular isoform. The identified *BuPAG* isoforms were classified into three separate groups of pregnancy namely, 45 days, 75 days and 90 days. The relative abundance of each BuPAG isoform at protein level was calculated as the percentage of total screened colonies in each group and the trend analysis was performed to analyze how the expression of identified BuPAG isoforms vary across the selected stages of pregnancy. The signal peptide prediction for all the identified isoforms was performed using online SignalP 4.0 server (http://www.cbs.dtu.dk/services/SignalP/) based on neural network trained on eukaryotes [45]. Physico-chemical properties were analyzed using Protparam server at ExPASy. The conserved domains were identified using PROSITE database at ExPASy [46]. The multiple alignments and percent identities among the identified BuPAGs and other reported PAGs in cow and buffalo were determined using Megalign module of DNASTAR software.

### Phylogenetic analysis

To study the evolutionary relationship of BuPAGs, the amino acid sequences of different bovine PAG isoforms (boPAG), PAG-like molecules and other mammalian aspartic proteinases were downloaded from NCBI with following accession numbers:boPAG 1 (AAB35845.1), boPAG 2 (NP_788787.1), boPAG 4 (NP_788788.1), boPAG 5 (AAC04678.1), boPAG 6 (AAC04679.1), boPAG 7 (ABV03360.1), boPAG 8 (AAC04681.1), boPAG 9 (NP_788793.1), boPAG 10 (AAC04683.2), boPAG 11 (AAC04684.1), boPAG 12 (AAC04685.1), boPAG 13 (AAF05996.1), boPAG 22 (AAX93317.1), PAG-like molecules i.e. bovine pepsin F (bovine: XP_002699356.2, human: NP_067428.2), chymosin (bovine: AAA30446.1, camel: NP_001290503.1, porcine: XP_020946458.1), pepsin (human: 1PSO_E, porcine: 3PEP_A, bovine: XP_019810172.1, goat: XP_005699798.1, sheep: XP_004019629.1), renin (bovine: XP_019832507.1, sheep: NP_001009299.1, mice: AAA40050.1, human: NP_000528.1), cathepsin D (human: NP_001900.1, bovine: XP_019810480.1, goat: XP_017898833.1, sheep: XP_012027348.1) and cathepsin E (bovine: XP_014338338.1, human: NP_001901.1). The sequences were aligned by Muscle program using default parameters [47]. The phylogenetic tree was constructed by MEGA6 software [48] using Neighbor-joining method (NJ) based on p-distance substitution model [49]. The reliability of clustering patterns was tested by calculating bootstrap support values (1000 replications).

### Homology modeling and molecular dynamic simulations of BuPAG 7

The 3D-structure of BuPAGs has not yet been reported; therefore, we chose to build the homology model of the most abundant isoform BuPAG7. The homology model was built using the YASARA molecular modeling package. YASARA uses the methods and protocols described by Krieger et al [50]. Briefly, PSI-BLAST iterations were run to search the PDB for potential modeling templates. The templates were ranked based on the alignment score and structural quality according to Whatcheck [51]. The alignments with the target sequence were obtained for each available template based upon the sequence-based profiles of target and template, structural information contained in the template and the predicted target secondary structure. During model building, optimization was done for possible loop conformations in case of any insertions or deletions, rotamer conformations and hydrogen bonding network. Subsequently, an unrestrained refinement was run using the knowledge-based force fields ensuring that the refinement did not move the model in the wrong direction. The steps were followed for all combinations of templates and alignments and quality indicators for the resulting models are calculated. Finally, a hybrid model was built by iterative replacements of bad regions in the top scoring model with corresponding fragments from the other models. If templates contained the ligands, various parameters associated with the molecular features were fully considered in the homology modeling procedure by YASARA, including hydrogen bonding and other interactions with the peptide chain.

The resultant hybrid model was subjected to further refinement to remove minor conformational strains using the md_refine.mcr macro of YASARA. Simulation parameters were kept at the values defined by the macro. Finally, the refined model was subjected to 30 ns constant temperature and pressure (NPT) molecular dynamics (MD) simulations using the md_run.mcr macro of YASARA. The structure of the protein was simulated in an 8 × 6 × 5 nm rectangular box with periodic boundaries and filled with a water density of 0.997 g/ml. The ion-concentration was kept at 0.9% NaCl. The trajectories were calculated with a time step of 2.5 fs using AMBER14 force-field and snapshots were saved at every 100 ps. The trajectories were analyzed using md_analyze.mcr macro. Ramachandran plot evaluation was done using PROCHECK through PDBSum server [52]. The docking protocol was set up and binding energy calculations were done by using molecular docking program VinaDock implemented in YASARA [53]. Structural analysis and superimpositions were done using YASARA and PyMOL 1.3.

## Results

High quality total RNA was isolated from buffalo fetal cotyledon tissue as evidenced from two distinct RNA bands corresponding to 18S and 28S. Reverse transcriptase PCR reaction of the total RNA was carried out under optimum conditions. The PCR amplification resulted in distinct bands of ~1230 bp size (S1 Fig).

### Identification, relative abundance and trend analysis of BuPAGs across early stages of pregnancy

In order to gain insight on the abundance of various isoforms during early pregnancy, the amplicons belonging to the early stages of pregnancy i.e. 45 days, 75 days and 90 days were cloned in *E*. *coli*. A total of 360 clones (120 clones from each stage) containing recombinant plasmids were used for sequencing.

In the 45 days pregnancy group, sequences of a total of 40 recombinant clones could be analyzed where 9 distinct BuPAG isoforms namely, BuPAG 2, 6, 7, 8, 9, 13, 15, 18 and one new BuPAG isoform (novel BuPAG) were identified (S2 Table). Out of 40 recombinant clones, 17 clones represented BuPAG 7 isoform, thus possessing a relative abundance of around 42%. Therefore, BuPAG 7 was found to be the most abundant isoform during 45 days pregnancy. The second most abundant isoform was BuPAG 18 (17.5%) which was followed by BuPAG 2, 8, 9, 15 and 13, respectively. One BuPAG sequence was found to share a maximum of 78% and 75% homology with the reported bovine PAG 6 and buffalo PAG 16, respectively. Looking at this low identity, it seems that it is an isoform which is distinct in our study (designated as novel BuPAG) and reported for the first time. However, the abundance of this isoform was very low (2.5%) and did not appear in other 2 stages of pregnancy. In the group representing 75 days of pregnancy, sequences of a total of 60 recombinant clones could be analyzed which revealed the presence of 7 BuPAG isoforms namely BuPAG 1, 2, 7, 13, 15, 16 and 18 in this group (S2 Table). BuPAG 1 and 16 were not detected in 45 days pregnancy group. BuPAG 7 was observed to be the most abundant isoform in this group also with a relative abundance of about 48%. The second most abundant isoforms were BuPAG 2 and 18 having a relative abundance of 13.3%. The frequency of BuPAG 18 isoform was relatively higher in 45 days pregnancy group. The frequency of BuPAG 2 seemed to increase slowly from 12% to 13%. We detected BuPAG 16 for the first time in this stage of pregnancy at a frequency of around 10%. BuPAG 1 was found to be the least abundant isoform in 75 days pregnancy group. In 90 days pregnancy group, sequences of a total of 9 BuPAG isoforms i.e. BuPAG 2, 4, 7, 8, 9, 13, 15, 16 and 18 were identified from 80 recombinant clones which could be analyzed. BuPAG 7 was found to be the most abundant isoform (41.25%) followed by BuPAG 18 (13.75) and BuPAG 8 (12.5) (S2 Table). We detected the expression of BuPAG 4 isoform in this group which was absent in previous stages.

**Fig 1:**
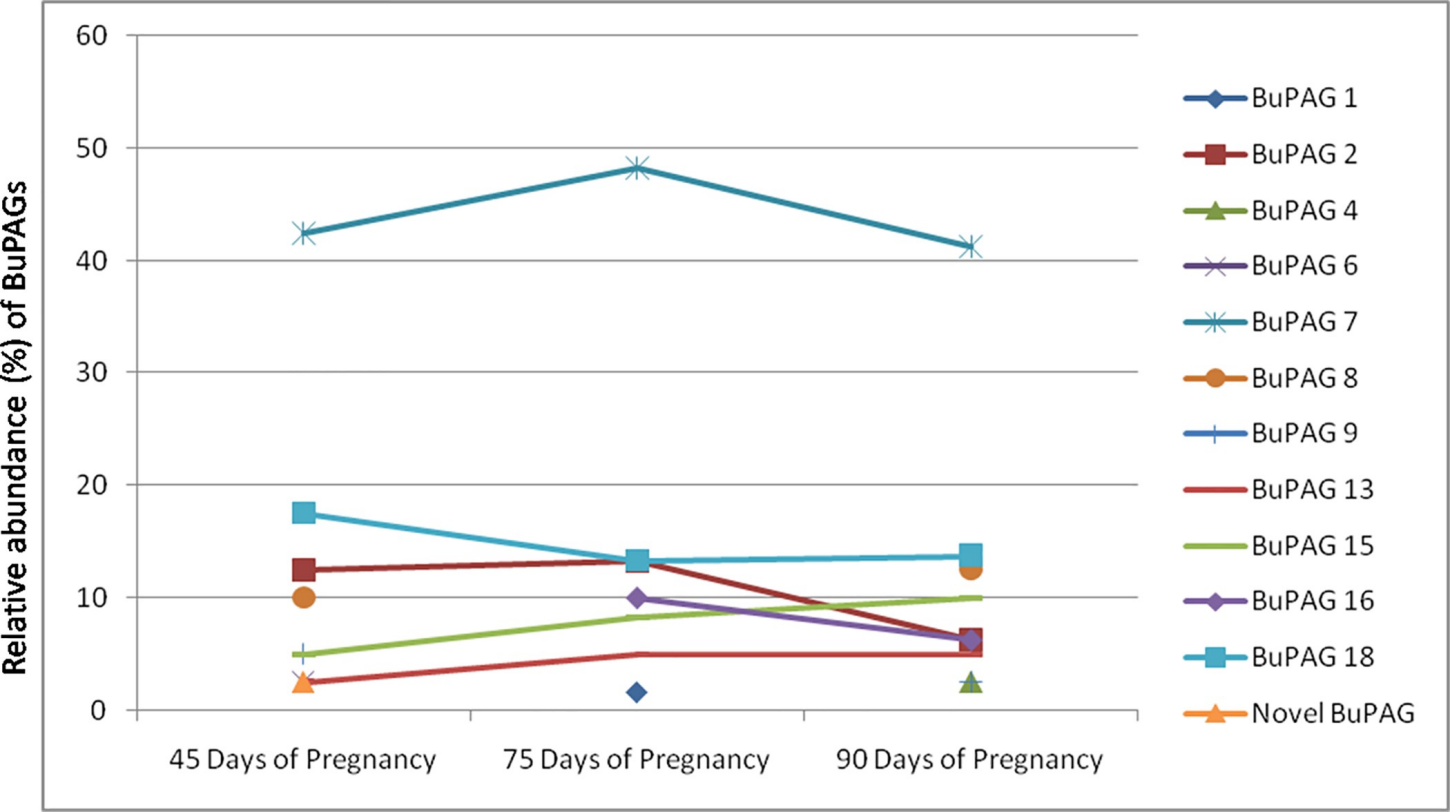
Trend analysis and relative abundance of BuPAGs. The figure shows the trend analysis of different BuPAG isoforms and their relative abundance across three stages of early pregnancy i.e. 45 days, 75 days and 90 days.

The trend analysis and relative abundance for appearance of different BuPAG isoforms are shown in Fig 1.The trend analysis indicated that BuPAG 7 was the most abundant isoform followed by BuPAG 18 as the second most abundant isoform which remained persistent in all 3 stages. BuPAG 6 was identified in 45 days pregnancy group, however, it was not observed in other groups. BuPAG 4 was observed for the first time in 90 days pregnancy group.

### Variants of BuPAG isoforms

The sequences for each identified isoform across three stages of pregnancy were aligned which revealed the existence of different variants for various isoforms. We detected 29 variants for the most abundant BuPAG 7 isoform sharing a homology of about 80% to 99%. Similarly, BuPAG 18 isoform observed in our study represented 11 variants. This was followed by BuPAG 8, 16 and 2 possessing 5, 4 and 2 variants, respectively. The percent identity matrices for different variants belonging to their respective isoforms are shown in S2 Fig. The nucleotide length and percent homology of various BuPAG isoforms and their respective variants with bovine and buffalo sequences reported in NCBI are shown in Table 1. The variant possessing the maximum sequence identity to either buffalo or bovine PAG sequence available at NCBI was used for the sequence characterization. The sequence characteristics of all identified variants are shown in S3 Table.

**Table 1:** Sequence characterization of various BuPAG isoforms and their variants.

S. No.	Isoforms	Nucleotide length (bp)	Length of ORF (bp)	Length (Amino acids)	% homology (aa) with BuPAG reported in NCBI	% homology (aa) with BoPAG reported in NCBI
1	BuPAG 1	1227	1143	380	100 (XP_006076402.1)	86 (XP_002699292.1)
2	BuPAG 2 variant 2	1215	1131	376	100 (ADO67790.1)	95 (NP_788787.1)
3	BuPAG 4	1227	1140	379	99 (XP_006076405.1)	96 (DAA13827.1)
4	BuPAG 6	1224	1140	379	91 (ADO67795.1)	88 (NP_788798.1)
5	BuPAG 7 variant 20	1227	1143	380	100 (ADO67796.1)	96 (AAI33470.1)
6	BuPAG 8 variant 3	1227	1143	380	99 (ADO67797.1)	93 (NP_788803.1)
7	BuPAG 9	1227	1143	380	99 (XP_006050181.1)	94 (NP_788793.1)
8	BuPAG 13	1227	1143	380	99 (ADO67802.1)	86 (XP_002699292.1)
9	BuPAG 15	1127	1143	380	99 (ADO67804.1)	94 (DAA13837.1)
10	BuPAG 16 variant 3	1227	1143	380	87 (ADO67805.1)	96 (DAA13834.1)
11	BuPAG 18 variant 4	1224	1140	379	100 (ADO67807.1)	97 (NP_001077166.1)
12	Novel BuPAG	1224	1140	379	79 (XP_006076403.1)	78 (DAA13834.1)

### Sequence analysis

**Fig 2:**
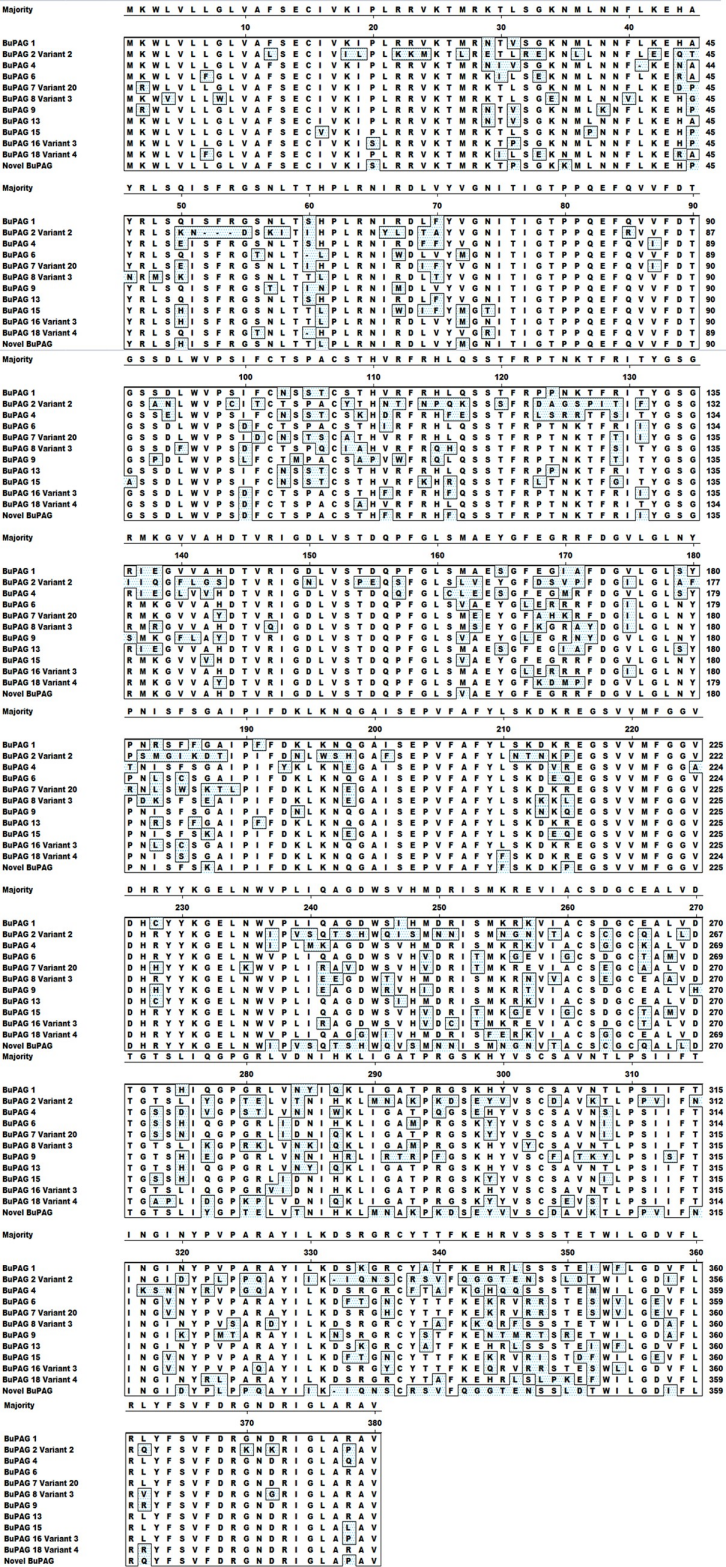
Multiple alignment: Sequence alignment of deduced amino acid sequences of different BuPAG isoforms. The residues in boxes differ from the consensus.

The multiple sequence alignment of deduced amino acid sequences of different BuPAG isoforms is shown in Fig 2. The alignment shows that isoforms possessed higher amino acid substitutions towards the c-terminal end of their sequences. Table 2 represents the physicochemical properties of all BuPAG isoforms based upon their deduced amino acid sequences. In all identified isoforms and their variants, a 15 amino acid long signal peptide was predicted and cleavage sites were positioned between the residues Cys 15 and Ile 16. The length of matured proteins ranged from 361 to 365 amino acids with molecular weights of ~40 kDa. It is noteworthy to mention that only BuPAG 2 isoform possessed an acidic pI of ~6.6 while other isoforms possessed basic pI ranging between ~9.2 to ~9.5. Furthermore, BuPAG 2 possessed the least number of positively charged residues (Arg+Lys) as compared to other isoforms. Sequence analysis revealed that all BuPAG isoforms are highly glycosylated and phosphorylated with various N-glycosylation (2–5), O- glycosylation (6–12) and phosphorylation (34–41) sites, respectively. Conserved eukaryotic and viral aspartyl protease motifs were identified in all BuPAG isoforms except BuPAG 8, 9 and 15, in which only one motif was found to be present either at N- or C- terminus.

**Table 2:** Physico-chemical properties of various BuPAG isoforms.

Isoform	Signal Peptide (Cleavage site)	Length of mature protein (AA)	Molecular weight (kDa)	Isoelectric point (pI)	Negatively charged residues (Asp + Glu)	Positively charged residues (Arg + Lys)	N-glycan sites	O-glycan sites	Phosphorylation sites	Viral and eukaryotic aspartyl protease motif I	Viral and eukaryotic aspartyl protease motif II
BuPAG 1	Cys 15-Ile 16	365	40.8	9.53	29	43	4	12	37	VVF**D**TGSSDLWV (86–97)	ALV**D**TGTSHIQG (267–278)
BuPAG 2 Variant 2	Cys 15-Ile 16	361	40.0	6.65	30	29	4	6	34	VVF**D**TGSANLWV(83–94)	ALL**D**TGTSLIYG (264–275)
BuPAG 4	Cys 15-Ile 16	364	40.7	9.26	32	42	3	6	41	VIF**D**TGSSELWV (84–95)	ALV**D**TGSSDIVG (266–277)
BuPAG 6	Cys 15-Ile 16	364	40.9	9.45	32	45	4	8	36	VVF**D**TGSANLWV(85–96)	AMV**D**TGSSHIQG (266–277)
BuPAG 7 Variant 20	Cys 15-Ile 16	365	41.2	9.64	33	50	4	8	40	VIF**D**TGSSDLWV (86–97)	ALV**D**TGSSNIQG(267–278)
BuPAG 8 Variant 3	Cys 15-Ile 16	365	40.9	9.63	32	49	3	6	36	VVF**D**TGSSDFWV(86–97)	-
BuPAG 9	Cys 15-Ile 16	365	41.1	9.69	27	44	3	8	41	VVF**D**TGSPDLWV (85–96)	-
BuPAG 13	Cys 15-Ile 16	365	40.8	9.53	29	43	4	10	34	VVF**D**TGSSDFWV(86–97)	ALV**D**TGSSNIQG(267–278)
BuPAG 15	Cys 15-Val 16	365	40.9	9.33	31	42	2	10	34	-	AMV**D**TGSSHIQG (267–278)
BuPAG 16 Variant 3	Cys15-Ile16	365	40.9	9.58	30	46	4	8	38	VVF**D**TGSSDLWV(86–97)	ALV**D**TGTSLIQG(267–278)
BuPAG 18 Variant 4	Cys15-Ile16	364	41.0	9.71	32	50	3	6	34	VVF**D**TGSSDLWV(85–96)	ALV**D**TGAPLIDG(266–277)
Novel BuPAG	Cys15-Ile16	364	40.7	9.23	29	39	5	10	35	VVF**D**TGSSDLWV(86–97)	ALL**D**TGTSLIYG((267–278)

### Evolutionary classification of BuPAGs

**Fig 3:**
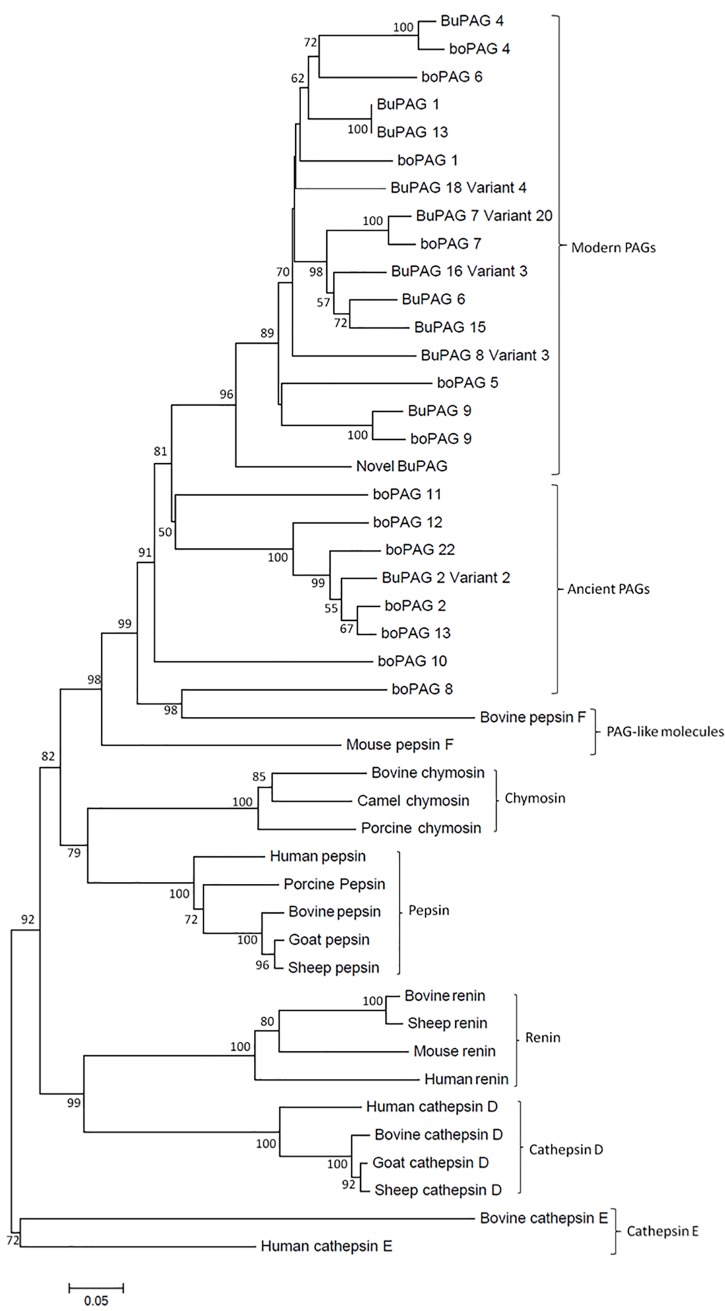
Evolutionary relationships among BuPAGs, bovine PAGs and other aspartic proteinases. The tree was created from the deduced amino acid sequences by the Neighbor Joining method in the MEGA 4.0 program. The tree was drawn to scale, and the numbers on the branches represent the confidence levels obtained from the bootstrap analysis (1000 replicates). The tree shows the separation of BuPAGs into two groups i.e. the modern and the ancient group.

Phylogenetic relationship among BuPAGs was analyzed and compared with PAGs in bovine, PAG- like molecules and enzymes belonging to aspartic proteinase family such as chymosin, pepsin, renin, cathepsin D and cathepsin E. For phylogenetic tree construction, many amino acid substitution models were applied where p-distance model showed reliable boostrap values for most of the important nodes. The tree suggests several gene duplication events in aspartic proteinase family leading to the segregation of various groups of paralogs. The duplication and divergence of the ancestral node led to the appearance of multiple forms of active and inactive aspartic proteases in mammals (Fig 3). PAGs in buffalo and bovine were found to be the closest to PAG-like molecules (pepsin F), chymosin, pepsin A and other aspartic proteinases.The initial duplication events showed the emergence of active peptidases cathepsin E, cathepsin D, renin, chymosin, pepsin and pepsin F. Cathepsin D and renin were grouped in a clade resulting from the duplication of an ancestral gene that gave origin to clades of other peptidases such as pepsin, chymosin and PAG-like molecules. Pepsin and chymosin form a sister group to the PAG-like molecules and PAGs. Subsequent duplications resulted in the evolution of pregnancy-associated glycoproteins which are further classified as ancient and modern PAGs (Fig 3 and S3 Fig). The branching pattern revealed that out of all identified BuPAGs in our study; eleven isoforms (BuPAG 1, BuPAG 4, BuPAG 6, BuPAG 7, BuPAG 8, BuPAG 9, BuPAG 13, BuPAG 15, BuPAG 16, BuPAG 18 and novel BuPAG) were grouped as modern PAGs which are generally considered as inactive. Only BuPAG 2 isoform belonged to the active ancient group of evolutionary classification.

### Structural modeling

**Fig 4:**
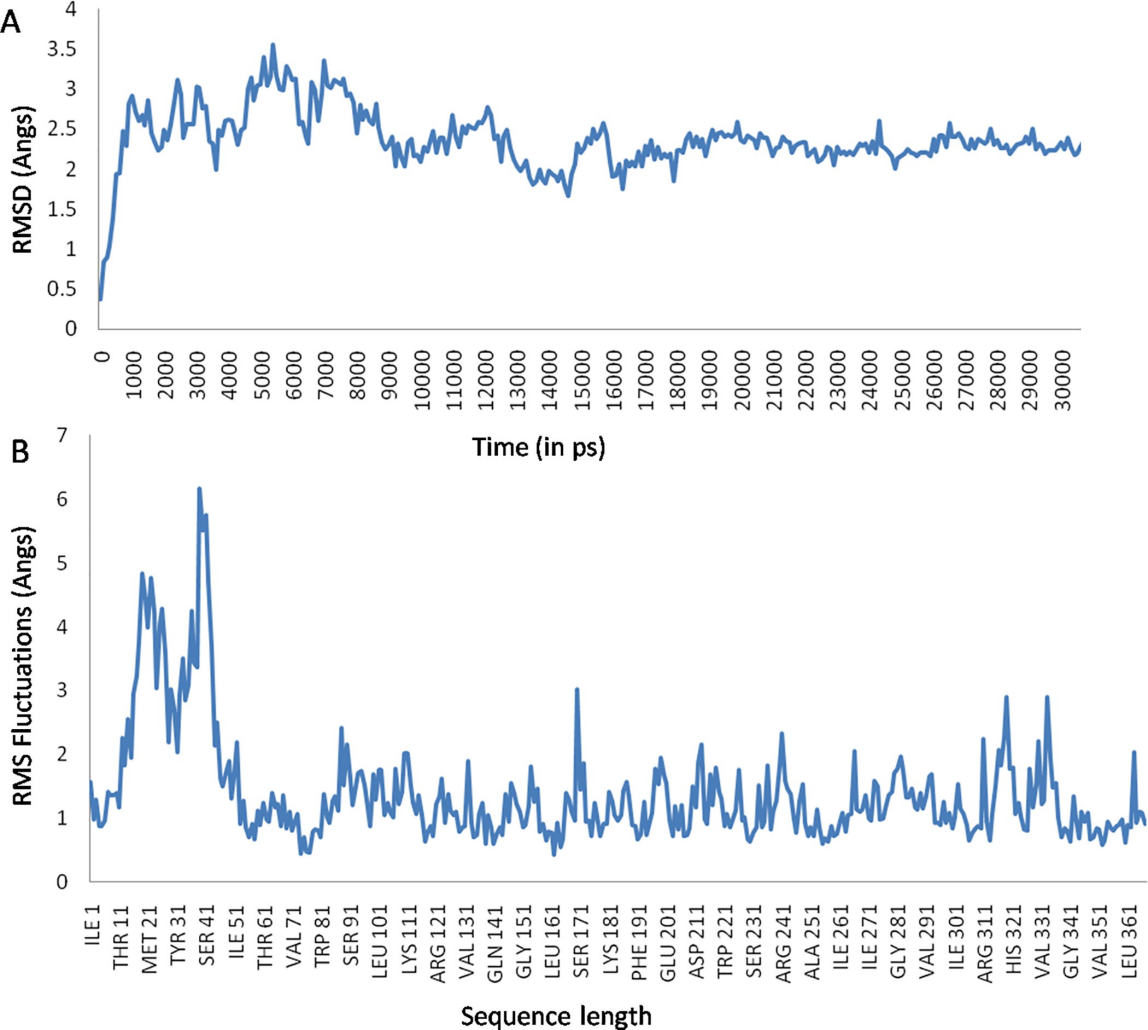
**RMSD and RMSF calculations for BuPAG 7 model structure (A)** Cα RMSD during 30 ns atomistic simulation run showing the structure stability and simulation integrity. **(B)** Observed RMSF per residue of BuPAG 7 over the 30 ns trajectory, which indicates the structural rigidity and measures the flexibility of the polypeptide chain. The region between 16±43 residues shows considerable flexibility.

**Fig 5:**
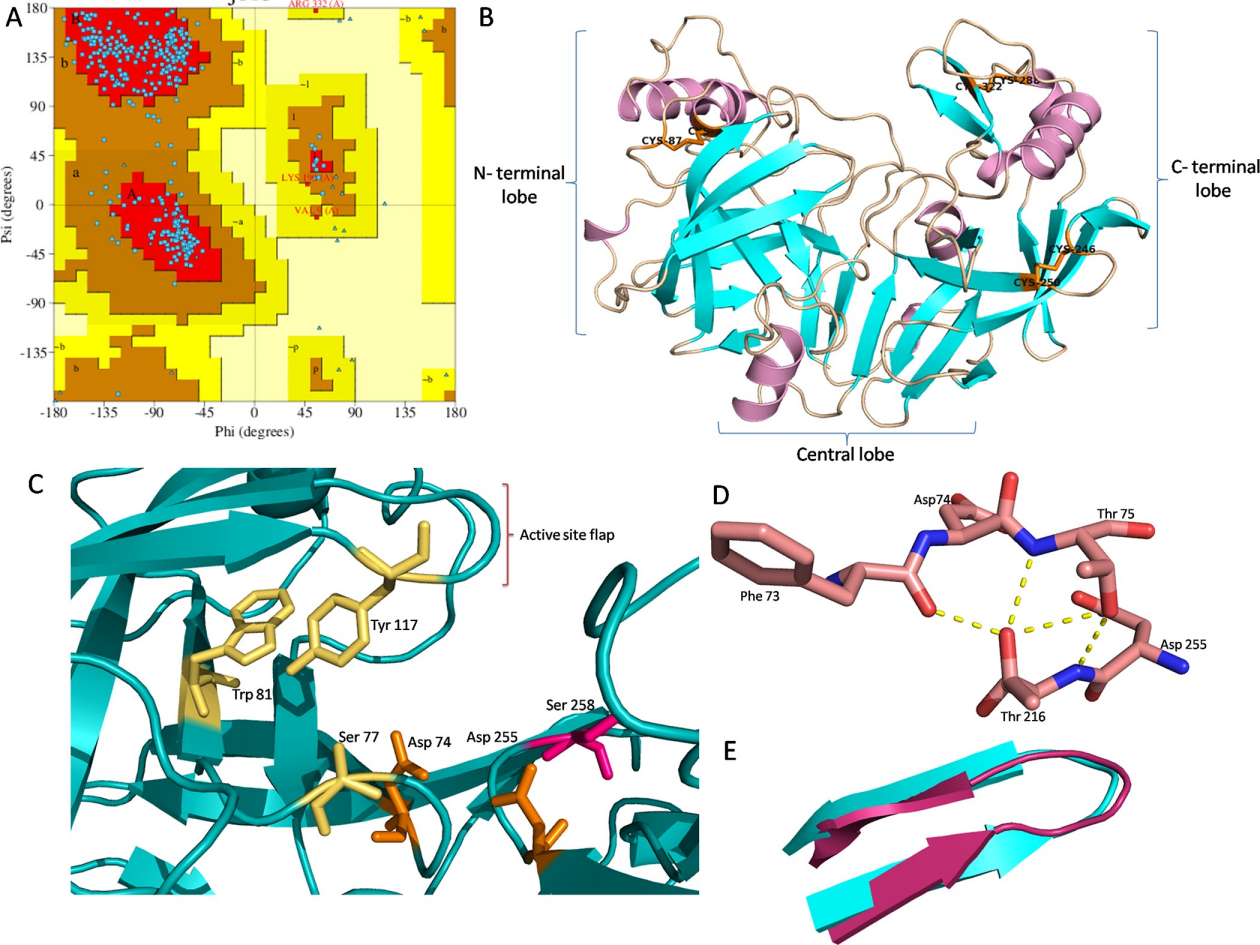
**(A) Ramachandran plot of the φ–ψ distribution of modeled BuPAG 7.** The red, brown and yellow regions represent the most favored, additionally allowed and generously allowed regions as defined by ProCheck. **(B) The overall structural display of BuPAG 7 in cartoon representation.** The figure shows N- and C- terminal lobes and a central domain connecting the two lobes. The substrate binding cleft is shown between the two lobes. α- helices are shown in purple and β- strands in cyan and connecting loops in the wheat color. Three disulphides (Cys 87- Cys 92, Cys 246- Cys 250 and Cys 288- Cys 322) are shown in orange sticks. **(C) The catalytic center of BuPAG 7.** The conserved key catalytic residues i.e. Asp 32 and Asp 215 (pepsin numbering) are shown in orange. Other conserved residues involved in the catalytic mechanism i.e. Ser 35, Trp 39 and Tyr 75 are shown in yellow. The single amino acid substitution i.e. Ser 218 in BuPAG 7 in place of Thr 218 in pepsin and other homologs is colored in pink. The active site flap over the catalytic site is indicated by the square bracket. **(D) Fireman’s Grip**. The conserved residues forming the Fireman’s Grip in BuPAG 7 for structural rigidity of catalytic center. **(E) Active site flap.** Superimposed active site flap of BuPAG 7 (cyan) over human pepsin (Magenta) showing a slight variation in the conformation of tip region.

Our study reveals that BuPAG 7 is the most abundant isoform among PAGs in the early stage of pregnancy; therefore, we attempted to unveil the structural features of this isoform to gain insights into its functional properties. BuPAG 7 consists of a total of 380 amino acids where first 15 residues are involved in the formation of the signal peptide. Therefore, the homology model was built for 365 amino acid long matured form of BuPAG 7. YASARA identified 10 possible templates after 5 PSI-BLAST iterations and target-template alignments were generated with 5 alignment variations per template for model construction. For each identified template, either a single model was constructed if the alignment was certain, or a number of alternative models if the alignment was ambiguous. A total of 46 models were sorted by their overall quality Z-scores. The Z-score has been defined as the weighted averages of the individual Z-scores using the formula, overall Z-scores = 0.145xDihedrals+0.390xPacking1D+0.465xPacking3D that describes how far away is the quality of model in terms of standard deviations from the average high-resolution X-ray structure [50]. The more negative values indicated that the homology model was worse than a high-resolution X-ray structure and the quality Z-score value below -2.0 was considered bad. The initial and topmost model was constructed with a Z-score of -0.415 on the basis of template 1PSO, possessing sequence coverage of 87.1%, sequence identity of 50.9% and sequence similarity of 67.6%, with BuPAG 7. Thereafter, to increase the accuracy of the constructed model, the best parts of the 46 models were combined, considering the initial model most suitable for hybridization, and a final hybrid model was generated with a quality Z-score of -0.756. 1PSO represents the crystal structure of human pepsin in complex with pepstatin; therefore, BuPAG 7 was modeled as a complexed structure with its inhibitor ligand pepstatin. The MD refinement by YASARA2 force-field generated 20 snapshots, one at every 25 ps; and the best snapshot was selected with a maximum quality score of -0.18 and minimum energy of -202805.19 kcal. The modeled complex structure of BuPAG 7 was further subjected to all atom MD simulations for 30 ns by optimizing its energy parameters and removing the structural strains. Large scale conformational changes were monitored through Root Mean Square Deviation (RMSD) measurements which showed the modeled structure to be in a stable phase (Fig 4A). The information about relatively rigid and flexible parts was attained by calculating the Root Mean Square Fluctuation (RMSF) per residue over the 30 ns trajectory (Fig 4B). It is the standard deviation of the atom position calculated from the average structure. The region covering the residues 16±43 was observed to be the most deviant. This region showed high RMSF thus suggesting its less ordered or highly flexible structural configuration. The stereochemical quality check of the fully refined model (1332 total residues) showed 90.6% residues falling in the most favored regions, 8.8% in the additional regions and 0.3% in the generously additional regions (Fig 5A). The model showed good stereochemical property in terms of the overall G-factor value of -0.14 calculated for dihedral angles and main chain covalent forces.

### Overall structure

The overall structure of BuPAG 7 was observed to be kidney- shaped and bilobal consisting of two N- and C- terminal lobes divided by a deep substrate- binding cleft. A central domain was observed to be positioned between N- and C- terminal lobes consisting of six antiparallel β- sheets which serve to act as a backbone to the active- site region of the molecule (Fig 5B). The six antiparallel β- sheets in central domain comprise residues 2–6, 189–194, 203–208, 214–224, 347–354, 357–365. Both N and C terminal ends of the molecule are positioned in the central domain. Secondary structural representation for BuPAG 7 model included 38.4% β- strands, 10.1% α- helix, 3.6% 3–10 helix and 47.9% other secondary structural elements. Three disulfide bonds were observed in the BuPAG 7 structure involving residues Cys 87- Cys 92, Cys 246- Cys 250 and Cys 288- Cys 322 (Fig 5B). No free cysteine was observed in the BuPAG 7 structure.

### Active site architecture of BuPAG 7

The catalytic residues Asp 32 and Asp 215 (pepsin numbering) are crucial for the catalytic activity of aspartic peptidases and are conserved in BuPAG 7. The corresponding residues in BuPAG 7 are Asp 74 and Asp 255 located at the center of the deep binding pocket formed above the central domain, and between the N-teminal and C-terminal lobes. In addition to these two aspartates, various other residues (Ser35, Trp39, Tyr75, and Thr218) are also involved in the catalytic mechanism of aspartic peptidases which contribute to form a continuous chain of hydrogen bonds which have been shown to be crucial for the catalytic activity by various protein engineering experiments [54]. The arrangement of these residues was found conserved in BuPAG 7 as that of other members of the aspartic peptidase family except for one amino acid substitution (Fig 5C). The conserved Thr 218 in pepsin with its hydrogen bond to Asp 215 in the active site is replaced by the corresponding Ser 258 in BuPAG 7. This Thr residue is conserved in other aspartic proteases including human pepsin, porcine pepsin and bovine chymosin. Though both the residues possess similar chemical properties, Ser 258 in BuPAG 7 lacks the ability to form a hydrogen bond to the catalytic Asp 255 due to different side chain orientation. In mouse renin, this Thr residue is also replaced by Ser 226 whereas in human renin, the corresponding residue is Ala229. An additional important network of hydrogen bonds, known as the "fireman's grip", was observed in the BuPAG 7 structure which stabilizes the active site of aspartic proteinases. The residues Thr 35, Phe 33 and Thr 217 are completely conserved in BuPAG 7 and make H bond network with Asp 32 and Asp 215 (Fig 5D).

Another significant conserved feature observed in the BuPAG 7 structure was a β- hairpin loop, also known as the active site “flap” (Fig 5E). The residues comprising this flap region range from residues 71–82 in human pepsin. The corresponding residues in the BuPAG 7 range from 113 to 124 where five amino acid substitutions were observed in comparison to human pepsin i.e. Val 71 to Phe 113, Ser 72 to Thr 114, Thr 74 to Ile 116, Thr 77 to Ser 119, Ser 79 to Arg 121 and Thr 81 to Lys 123. The replacement of flap residues with residues of different physicochemical properties does not seem to alter the flap conformation except for some slight variations in tip region; however, they may serve to attribute specific binding properties to BuPAG 7.

### Substrate-binding pocket and interactions with pepstatin

The substrate binding pocket of BuPAG 7 revealed that most residues crucial for substrate binding and catalysis were conserved. When compared to human and porcine pepsin, the residues Asp 32, Gly 74, Tyr 75, Gly 76, Ile 120, Asp 215, Gly 217, Ser 215 (pepsin numbering) were conserved in the substrate binding pocket of these enzymes including BuPAG 7. However, variations were observed in various other substrate binding residues with nine amino acid substitutions when compared to human pepsin and seven substitutions when compared to porcine pepsin (Table 3). The replacement of these residues with residues of significantly different physicochemical properties may have implications on the shape, size, charge and binding properties of the pocket.

**Table 3:** Amino acid substitutions in substrate binding cleft of BuPAG 7 in comparison to human pepsin and porcine pepsin.

S. No.	BuPAG 7	Human Pepsin	Porcine Pepsin
1	Ile 54	Met 12	Thr 12
2	Ile 72	Val 30	-
3	Ile 116	Thr 74	Thr 74
4	Ser 119	Thr 77	Thr 77
5	Asp 229	Tyr 189	Tyr 189
6	Ser 258	Thr 218	Thr 218
7	Lys 327	Gln 287	Glu 288
8	Lys 329	Met 289	Met 290
9	Val 331	Leu 291	-

**Fig 6:**
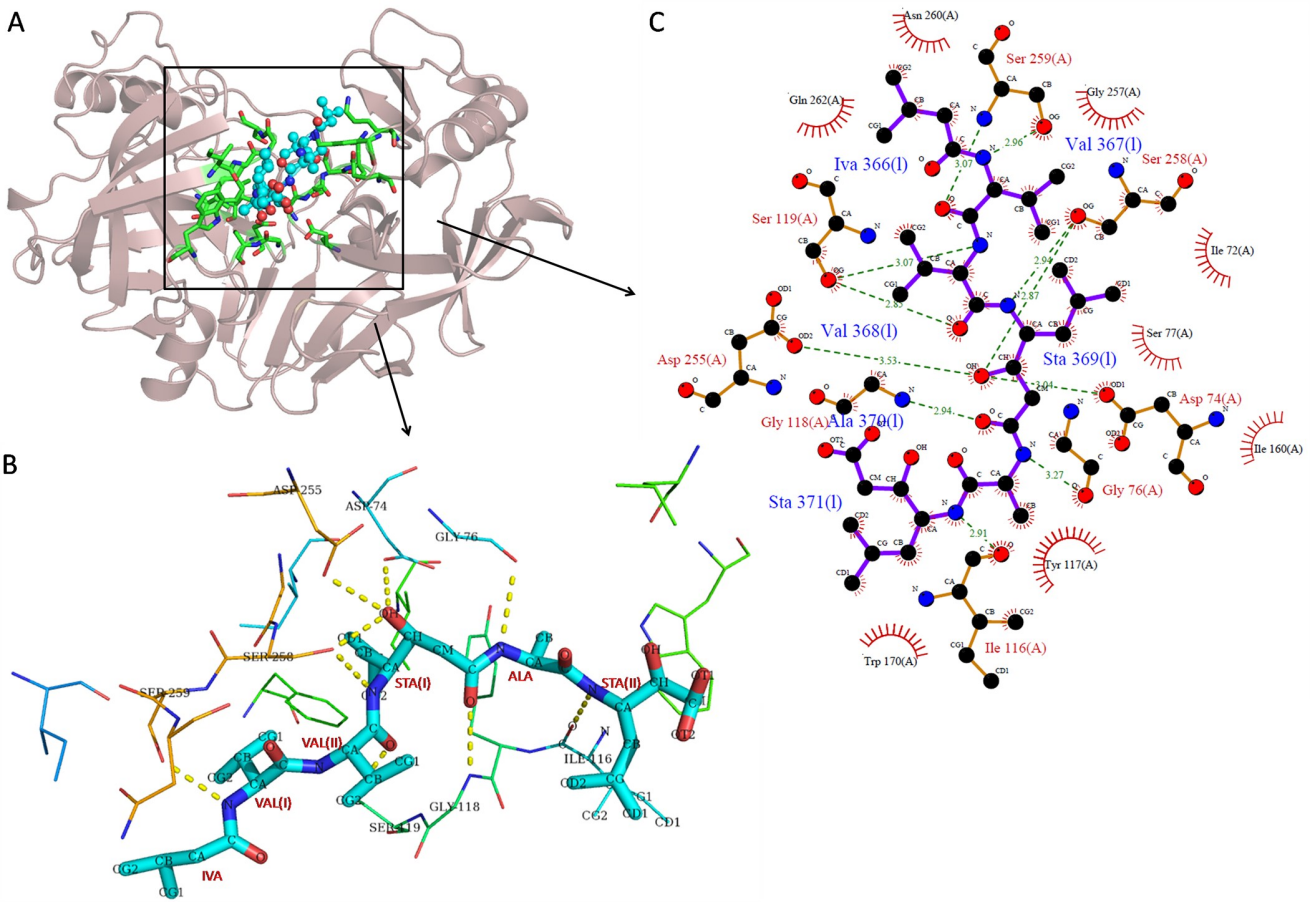
Substrate-binding pocket and interactions of BuPAG 7 with pepstatin. **(A)** The overall structure of BuPAG 7 in complex with pepstatin in its substrate-binding pocket. Pepstatin is shown in ball and sticks representation (cyan) and interacting residues in sticks representation (green). **(B)** Residues involved in hydrogen bonding and other interactions with the side chains of pepstatin in sticks representation (cyan). **(C)** A schematic representation of interactions of pepstatin with BuPAG 7 generated using ligplot. Interactions involve both hydrogen bonding and hydrophobic contacts. Hydrogen bonds are indicated by dashed lines (green) between the atoms involved, while hydrophobic contacts are represented by an arc (red) with spokes radiating towards the ligand atoms they contact. The contacted atoms are shown with spokes radiating back.

**Fig 7:**
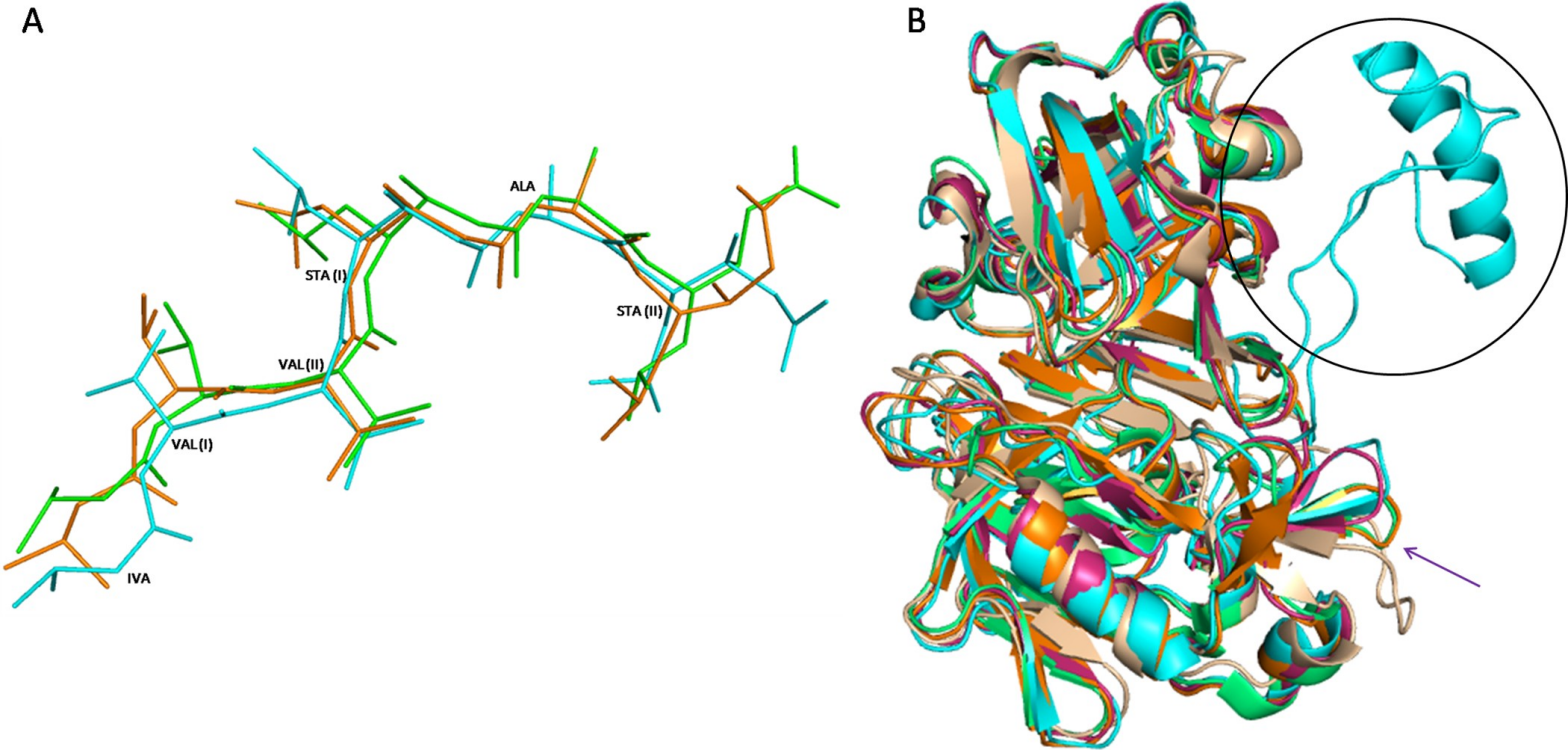
Structural comparison with other aspartyl proteases. **(A)** Superposition of pepstatin structures bound to BuPAG 7 (cyan), human pepsin (orange) and bovine chymosin (green). Pepstatin adopts an almost similar conformation in BuPAG 7 except for the extreme ends. **(B)** The superimposed structures of BuPAG 7, Human pepsin (1PSO), porcine pepsin (4PEP), bovine chymosin (4AUC) and human renin (3D91). The longer insertion region (35–41) in BuPAG 7 protruding out of the N-terminal lobe as compared to others is shown in circle. The change in conformation of a shorter loop is indicted by an arrow.

The substrate binding pocket in BuPAG 7 was sufficiently deep and extended in order to accommodate a seven residue long pepstatin inhibitor. Fig 6A shows the complex structure of BuPAG 7 with pepstatin inhibitor in its substrate binding pocket. Pepstatin adopts an extended conformation, as observed for other aspartic proteinases, with the first statyl hydroxyl oxygen occupying a position in the active site and forming a hydrogen bond to the catalytic Asp 74 (Fig 6B). The residues involved in hydrogen bonding and making Vander Waals contact with the side chains of pepstatin are shown in Fig 6C. The structures of pepstatin bound to BuPAG 7, human pepsin (1PSO), and bovine chymosin (4AUC) have been superposed in Fig 7A. It shows that the inhibitor binds in a similar conformation to all enzymes except for the extreme end with the second statyl which might be due to more flexibility of this residue.

The average binding energy and dissociation constant of 7.4 kcal/mol and 4.0 μM were calculated for BuPAG 7-pepstatin complex. In the case of pepsin-pepstatin complex, the calculated binding energies were similar for human pepsin 1PSO (-6.02 kcal/mol) and bovine chymosin 4AUC (-5.87 kcal/mol). This indicates that, like other aspartic peptidases, BuPAG 7 has a stronger binding affinity with pepstatin as shown by the calculated binding energy. Therefore, the residue substitutions in the substrate binding pocket with residues of different nature suggests that either the chemical nature of these residues might not be very crucial for substrate binding or they have evolved for a different subset of receptors or ligands.

### Structural comparison with other aspartyl proteases

The alignment of BuPAG 7 with other aspartyl proteases revealed maximum sequence identity with Human Pepsin (50%) followed by Porcine Pepsin (47.83%), Bovine Chymosin (42.19%) and Human Renin (33.54%). Structural superpositions of BuPAG 7 model revealed RMSD values of 0.907 with Human Pepsin (1PSO) (for 279 Cα atoms), 1.057 with Porcine Pepsin (4PEP) (for 278 Cα atoms), 1.099 with Bovine Chymosin (4AUC) (for 270 Cα atoms), and 1.249 with Human Renin (3D91) (for 270 Cα atoms), respectively. This suggests that in spite of the presence of various amino acid substitutions, BuPAG 7 displays an overall fold similar to those of the above structures except for some deviations. One distinct and striking feature observed was the presence of a 35–41 amino acid long insertion forming a part of the N- terminal lobe of BuPAG 7 structure in comparison to other aspartic proteases (Fig 7B). The insertion was found to consist of Lys14-Phe55, Thr11-Arg52, Leu16-Phe55, and Ser17-Arg52 in human pepsin, porcine pepsin, bovine chymosin and human renin, respectively. The insertion region is represented by an alpha helix (Asn20-Gln35) which is connected by two loops. Another difference observed was the presence of a longer loop (Phe286 to Leu295) in human renin as compared to BuPAG 7, human pepsin, porcine pepsin and bovine chymosin. This stretch of residues was found to be deleted in BuPAG 7, human pepsin, porcine pepsin and bovine chymosin. Though the size of this shorter loop in BuPAG 7 was almost similar to human pepsin, porcine pepsin and bovine chymosin, the conformation was most similar to that of human pepsin. The presence of these insertions and deletions away from the substrate binding site and the active site suggests that they might not be very crucial for the ligand binding properties of BuPAG 7 or they have appeared for a different set of functions.

## Discussion

In the present investigation, we have identified and characterized different PAG isoforms and their variants during early stages of pregnancy in buffalo. To our knowledge, this is the first study to report different isoforms and their variants in buffalo on their relative abundance, evolutionary relationship and other sequence properties. The study also reports various conserved and unique structural features of the most abundant isoform BuPAG 7 by modelling approach. Although few reports are available on BuPAGs and specifically the PAG 1 and PAG 2 genes [40, 42, 43], the information on the existence of other isoforms and variants of BuPAGs is not well addressed. Also, there is a lack of an in-depth analysis of the previously reported model structures (PAG 1 and PAG 2). A large number of PAGs have been identified in bovine and ovine species, at transcript level and their existence has been confirmed using either through the preparation of cDNA library or northern blot. In these species, these isoforms (more appropriately, the mRNA) have been named sequentially upon identification. The approximate number of probable transcripts is proposed to be in order of more than 100 [13, 55]. In buffalo, the screening for similar PAGs has been carried out by other research groups and their partial sequences have been deposited in GenBank at NCBI [4]. They have reported a total of around 21 transcripts throughout pregnancy. We tried to observe the pattern of expression of these transcripts during the early phase of pregnancy (i.e. below 45 days of pregnancy till 90^th^ day). It has been reported that placentation starts at around day 25 of gestation in cattle [31]. We tried to isolate 30 days old cotyledon-like structures for isolation of RNA. As an alternate approach, we attempted to isolate RNA from the whole placental membrane from less than 30 days pregnant animal with tiny cotyledon-like structures. However, buffalo-specific PAG transcripts could not be amplified suggesting that there may be variability in the expression of the PAGs in buffalo as compared to cattle. A large number of cDNA clones were screened with the assumption that individual transcripts will be represented in clones based on their relative abundance of expression in the early time window of pregnancy. To avoid any biological variation in the pattern of expression of different PAG isoforms, four separate animals were taken at each time points i.e. 45 days, 75 days and 90 days of pregnancy. We compared our sequences with both the bovine and bubaline PAG databases and found that there is non-coherence in the nomenclature of the different PAG isoforms. Therefore, we decided to use the naming of individual transcripts based on its maximum homology with a particular isoform between bovine and bubaline. Thus, we could identify a total of 12 distinct transcripts in the time window of 45 to 90 days of pregnancy.

Studies have indicated that there are temporal and spatial differences in the expression of various isoforms of PAGs in bovine [4, 28, 56]. In our study, the BuPAGs isoforms 7, 18, 2, 8, 6, 9, 13 and 15 were detected at early stage. Other research groups have reported that there is a stage-specific expression of PAGs [4, 15, 26]. The PAG molecules which were predominantly expressed in the binucleate cells of cattle (e.g. BoPAG 1, 6, and 7) were expressed weakly or not expressed in 25 days old cotyledons but they were present at the middle and late stages of pregnancy. Other isoforms e.g. BoPAG 4, 5 and 9 were observed around day 25 and earlier. In another study, cDNA microarray analysis demonstrated that several PAG molecules are expressed as early as day 7 to 14 of pregnancy (BoPAG 11, 16 and 17), day 14 to 21 (BoPAG 1, 5 to 7, 9 to 13, 15 to 17, 19, 21) or even before (at day 7: BoPAG 4, 5 and 6) [57]. In one of our recent cDNA microarray study, different PAGs in buffalo (PAG 4, 5, 6, 7, 8, 11 and 17) were identified as top-upregulated proteins by cDNA microarray analysis in fetal cotyledons during early pregnancy [58]. It has been reported that in species with epitheliochorial placentation, PAGs (equine: eqPAG 1, porcine: poPAG 1, and poPAG 2) are expressed throughout the chorion [12, 40]]. From quantitative expression point of view, the relative expression of BuPAG 7 was the highest among all isoforms in buffalo In contrast, bovine BoPAG 2 is the most abundant transcript among all the PAGs identified in cattle and BoPAG 12 is the least prevalent isoform in cattle differing by as much as two to three orders of magnitude at any given time-point during pregnancy [18] suggesting the species-specific preferential expression of transcripts.

Our finding for the expression of many variants for individual isoforms raises questions whether these identified copies of genes are present on buffalo genome or they are the aberrant transcription products of the same gene. Genomic DNA of placental cells (specifically cotyledonary cells) is the result of a rapid division of nucleus leading to the formation of binucleate cells. It was reported in a study that around 20–30% of trophoblastic cells are binucleate [59]. It seems quite possible that a large number of variants in mRNA transcripts may be arising because of simultaneous rapid replication and transcription. However, the existence of multiple genes for all these variants on genome cannot be overruled. Southern blotting with exon-specific probes showed that many genes related to both PAG 1 and PAG 2 are present in cattle, sheep and related ruminants [3, 13–15]. Twenty-one distinct full-length cDNA representing BoPAG members have been cloned from cattle but there is a likelihood of occurrence of many more isoforms or variants [4]. We observed in our study that the isoforms which are relatively more abundant also have relatively more transcript variants. A possible explanation behind a large number of variants and isoforms (gene family) is probably due to local selection pressure on the development of chorionic trophoblastic cells. These cells lie at the interface of endometrium and fetus where they perform a range of transport and endocrinological functions that provide support to the developing fetus [18]. The physiological demands of the fetus and the response of mother towards these demands create a local selection pressure at the site of interface [60]. Different PAGs (many isoforms and variants) with different specificities and affinities for their substrates might be helping in smaller peptide transport from mother to fetus. It is yet to be tested whether all these transcripts are translated into protein or not, as the evidence of BuPAG isoforms at the protein level is very less [4]. Looking at the large number of variants among these isoforms, it can be assumed that the rate of replication is very fast in trophoblastic cells (especially the binucleate cells) which cannot maintain the fidelity of replication. Therefore, the transcripts resulting from such aberrant DNA result in a large number of variants. Such infidelity doesn’t pose any problem as they are not going to be a part of either the fetus or mother. It is noteworthy to mention that only a few variants have been observed for few PAG isoforms in bovine (BoPAG 2, 3, 7, 8, 14, 15 etc.) (NCBI GenBank database). A large number of transcript variants for individual isoforms indicate the possibility of the existence of many more such variants. The variability in PAG sequence may be associated with transcriptionally active regions in the genome with high rate of replication. This assumption goes in consonance with the previous findings that almost 20% of trophoectodermal cells divide rapidly to form binucleate cells which cannot follow cytokinesis [61]. Clearly the significance of such alteration in genome will not be permanent as these binucleate cells die after fusion with maternal epithelial cells forming a transient trinucleate structure which is supposed to serve as a factory for the production of necessary molecules for the maintenance of pregnancy.

### Evolution of BuPAGs and their functional significance

On the evolutionary scale, bovine PAGs are grouped into two categories: ancient (which evolved > 87 million years ago) and modern (≤ 52 million years ago) [4, 62]. The members of ancient group of PAGs are transcribed in all cotyledonary trophoblast cell types while the PAGs belonging to the modern group are transcribed exclusively by a specialized subset of trophoblasts called binucleate cells (BNC [4, 15, 28, 62]. Usually in cattle, PAGs 2, 8, 10, 11, 12 and 13 have been classified as ancient PAGs [18]. The prototypic members from these families in bovine are PAG 2 and PAG 1 in ancient and modern group respectively. In contrast to this, out of 12 identified BuPAG isoforms in buffalo, only BuPAG 2 is classified as ancient PAG in our study while others are similar to the members of the modern PAG group. It is further supported by our observation that only BuPAG 2 isoform belonging to ancient group possessed an acidic pI while all other isoforms belonging to the modern group were found to possess basic pI. Thus, it may be inferred that modern PAGs have evolved to perform some specific functions by interactions with various biomolecules. Furthermore, the structure of aspartic proteinases is proposed to have arisen by internal gene duplications with N-terminal and C-terminal lobes including a central lobe [63, 64]. This provides an evidence of homology between these aspartic proteinases including BuPAGs. The phylogenetic inference in our study further reveals the close relationship of PAGs and PAG-like molecules to these aspartic proteinases.

### Structure-based function of BuPAGs

Historically, PAGs have been called as pregnancy-associated. But, it is important that these proteins should be explained with respect to their functions. The existence of a specific BuPAG isoform may have a significant role in the maintenance of pregnancy and serving important functions which are not yet clear. In our study, BuPAG 7 was found to be the most abundant isoform which consisted of more than 40% of the total transcripts. Therefore, we deciphered the structure of BuPAG 7 so that a possible functional significance of this isoform could be explained. Structural modeling has shown that BuPAG 7 retains the well- known bilobed structure of aspartic proteinases with a peptide binding cleft between the two lobes. The three-dimensional fold of BuPAG 7 is very similar to other aspartic proteinases, however, the amino acid sequences seem to be more divergent, except for the conserved catalytic site motif. BuPAG 7 is assumed to be enzymatically inactive due to key mutations within the binding cleft; however, reports have suggested that PAGs can bind to pepstatin A which is a powerful inhibitor of aspartic proteinases [38, 56]. Thus, it can be concluded that such overexpressing molecule either has a role to play in pregnancy which is different from aspartic proteases or the enzymatic role of this molecule needs to be tested further. The two lobes of the molecule contribute two aspartic residues in the catalytic site. The protein is also characterized by the presence of disulfide bridges at conserved positions like other aspartic peptidases which have been proposed to impart integrity and stability to the structure [65, 66]. The Tyr 75 residue (pepsin numbering) hanging from the tip of the active site flap has been suggested to be crucial for the catalytic mechanism in aspartic peptidases [63]. Tyr at 117^th^ position in BuPAG 7 is found conserved. Various amino acid substitutions were also observed in BuPAG 7 structure which may be responsible for specific biological functions of this isoform.

## Conclusions

This is the first study which reports 12 BuPAG isoforms and a total of 56 variants during early stages of pregnancy with full sequence information. BuPAG 7 was found to be the most abundant isoform with a maximum number of variants detected at transcript level as early as less than 45 days which remained elevated till 90 days of pregnancy. All buffalo PAGs were found to have pI typical of modern PAGs indicating that these PAGs have evolved to perform a unique function in a different microenvironment. Further, the overall structure of BuPAG 7 was observed to be similar to aspartic proteinase family members. In spite of the presence of various amino acid substitutions in the substrate binding site, pepstatin binds to BuPAG 7 in a similar conformation to that of other aspartic proteinases. The dairy industry is in need of a pregnancy biomarker for economically important livestock species like buffalo. Therefore, PAG 7 can be predicated as a potential biomarker for early detection of pregnancy in buffalo. The function of none of the PAGs has been proven to date. In future, elucidation of the structure and function of ancient and modern PAGs may reveal important information on reproductive biology.

## Supporting information

S1 Table Sequence of primers targeting PAG genes. (DOCX)

S2 Table BuPAG isoforms at around 45 days, 75 days and 90 days of pregnancy. (DOCX)

S3 Table Sequence characterization of various BuPAG isoforms and their variants. (DOCX)

S1 Fig **Total RNA and amplified cDNA representative of three stages of pregnancy i.e. 45 days, 75 days and 90 days: A.** Total RNA isolated from cotyledonary tissue at 45 days (lane 1), 75 days (lane 2) and 90 days (lane 3) separated on 1.2% agarose gel representing 28S and 18S intact rRNA bands. **B.** Agarose gel electrophoresis of PCR amplified BuPAG genes. PCR product of size ~1.2 kb was observed at 45 days (lane 1), 75 days (lane 2) and 90 days (lane 3) pregnancy. Lane 4 represents the DNA ladder.
(DOCX)

S2 Fig **Percent identity matrices of variants belonging to (A)** BuPAG 2 **(B)** BuPAG 7 **(C)** BuPAG 8 **(D)** BuPAG 16 **and (E)** BuPAG 18.
(DOCX)

S3 Fig Evolutionary relationships among different isoforms of BuPAGs and their variants: The tree was created from the deduced amino acid sequences by the Neighbor Joining method in the MEGA 4.0 program. The tree was drawn to scale, and the numbers on the branches represent the confidence levels obtained from the bootstrap analysis (1000 replicates).
(DOCX)
