# Supplementary material for: Primary structures of different isoforms of buffalo pregnancy-associated glycoproteins (BuPAGs) during early pregnancy and elucidation of the 3-dimensional structure of the most abundant isoform BuPAG 7
Source: PLoS One. 2018 Nov 7;13(11):e0206143. doi: 10.1371/journal.pone.0206143 (PMC6221303; doi:10.1371/journal.pone.0206143)
Supplement: S1 Table — (DOCX) [file pone.0206143.s001.docx]

**S1 Table:** Sequence of primers targeting PAG genes

| **Name/ Label** | **Sequence 5´ to 3´** | **Product size (approximate nucleotides)** | **Tm value** |
| --- | --- | --- | --- |
| **PCR Primers** | | | |
| BoPAG Fcon | G AGC CAG GAA AGA AGC ATG | 1230 | 62.5 |
| BoPAGF 1 | CT TGG A**T**C CAG GAA A**T**C A**A**C ATG | 1230 | 63.1 |
| BoPAGF 4 | CT TGG AGC CAG GAA AGA A**A**C | 1230 | 62.6 |
| BoPAGF 3 | CT TGG AGC **T**AG GAA A**T**A **CA**C ATG | 1230 | 61.4 |
| BoPAGF 5 | CT TGG AGC CAG GAA AGA AG**T** | 1230 | 62.3 |
| BoPAG Rcommon | GAG TGC CCA AAG TGT GAG TGA | - | 65.3 |
| **Sequencing primers** | | | |
| T7 Forward | CGACTCACTATAGGGAGAGCGGC | - | - |
| pJET1.2 Reverse | AAGAACATCGATTTTCCATGGCAG | - | - |
