# Supplementary material for: Primary structures of different isoforms of buffalo pregnancy-associated glycoproteins (BuPAGs) during early pregnancy and elucidation of the 3-dimensional structure of the most abundant isoform BuPAG 7
Source: PLoS One. 2018 Nov 7;13(11):e0206143. doi: 10.1371/journal.pone.0206143 (PMC6221303; doi:10.1371/journal.pone.0206143)
Supplement: S2 Table — (DOCX) [file pone.0206143.s002.docx]

**S2 Table:** BuPAG isoforms at around 45 days, 75 days and 90 days of pregnancy

|  |  | **45 Days of Pregnancy** | **75 Days of Pregnancy** | **90 Days of Pregnancy** |
| --- | --- | --- | --- | --- |
| **S.No.** | **Isoform** | **% Frequency (Out of total 40 sequences)** | **% Frequency (Out of total 60 sequences)** | **% Frequency (Out of total 80 sequences)** |
| 1 | BuPAG 1 | - | 1.6 | - |
| 2 | BuPAG 2 | 12.5 | 13.3 | 6.25 |
| 3 | BuPAG 4 | - | - | 2.5 |
| 4 | BuPAG 6 | 2.5 | - | - |
| 5 | BuPAG 7 | 42.5 | 48.3 | 41.25 |
| 6 | BuPAG 8 | 10.0 | - | 12.5 |
| 7 | BuPAG 9 | 5.0 | - | 2.5 |
| 8 | BuPAG 13 | 2.5 | 5.0 | 5.0 |
| 9 | BuPAG 15 | 5.0 | 8.3 | 10.0 |
| 10 | BuPAG 16 | - | 10.0 | 6.25 |
| 11 | BuPAG 18 | 17.5 | 13.3 | 13.75 |
| 12 | Novel BuPAG | 2.5 | - | - |
