# Supplementary material for: Primary structures of different isoforms of buffalo pregnancy-associated glycoproteins (BuPAGs) during early pregnancy and elucidation of the 3-dimensional structure of the most abundant isoform BuPAG 7
Source: PLoS One. 2018 Nov 7;13(11):e0206143. doi: 10.1371/journal.pone.0206143 (PMC6221303; doi:10.1371/journal.pone.0206143)
Supplement: S3 Table — (DOCX) [file pone.0206143.s003.docx]

**S3 Table:** Sequence characterization of various BuPAG isoforms and their variants

| **S. No.** | **Isoforms** | **Transcript variants** | **Nucleotide length (bp)** | **Length of ORF (bp)** | **Length (Amino acids)** | **% homology (aa) with Buffalo PAG reported in NCBI** | **% homology (aa) with Bovine PAG reported in NCBI** |
| --- | --- | --- | --- | --- | --- | --- | --- |
| 1 | BuPAG 1 | | 1227 | 1143 | 380 | 100  (XP_006076402.1) | 86  (XP_002699292.1) |
| 2 | BuPAG 2 | BuPAG 2 variant 1 | 1215 | 1131 | 376 | 99 | 94 |
|  |  | BuPAG 2 variant 2 | 1215 | 1131 | 376 | 100 (ADO67790.1) | 95 (NP_788787.1) |
|  |  | BuPAG 2 variant 3 | 1215 | 1131 | 376 | 99 | 94 |
|  |  | BuPAG 2 variant 4 | 1073 | 897 | 298 | 99 | 85 |
| 3 | BuPAG 4 |  | 1227 | 1140 | 379 | 99  (XP_006076405.1) | 96  (DAA13827.1) |
| 4 | BuPAG 6 |  | 1224 | 1140 | 379 | 91  (ADO67795.1) | 88  (NP_788798.1) |
| 5 | BuPAG 7 | BuPAG 7 variant 1 | 1228 | 1143 | 380 | 98 | 94 |
|  |  | BuPAG 7 variant 2 | 1227 | 1143 | 380 | 87 | 95 |
|  |  | BuPAG 7 variant 3 | 1227 | 1143 | 380 | 88 | 93 |
|  |  | BuPAG 7 variant 4 | 1227 | 1143 | 380 | 87 | 96 |
|  |  | BuPAG 7 variant 5 | 1227 | 1143 | 380 | 99 | 96 |
|  |  | BuPAG 7 variant 6 | 1227 | 1143 | 380 | 99 | 97 |
|  |  | BuPAG 7 variant 7 | 1227 | 1143 | 380 | 90 | 87 |
|  |  | BuPAG 7 variant 8 | 1227 | 1143 | 380 | 88 | 94 |
|  |  | BuPAG 7 variant 9 | 1227 | 1143 | 380 | 87 | 94 |
|  |  | BuPAG 7 variant 10 | 1227 | 1143 | 380 | 92 | 91 |
|  |  | BuPAG 7 variant 11 | 1110 | 1026 | 341 | 89 | 96 |
|  |  | BuPAG 7 variant 12 | 1227 | 1143 | 380 | 95 | 92 |
|  |  | BuPAG 7 variant 13 | 1227 | 1143 | 380 | 95 | 92 |
|  |  | BuPAG 7 variant 14 | 1227 | 1143 | 380 | 88 | 94 |
|  |  | BuPAG 7 variant 15 | 1227 | 1143 | 380 | 87 | 95 |
|  |  | BuPAG 7 variant 16 | 1224 | 1140 | 379 | 96 | 94 |
|  |  | BuPAG 7 variant 17 | 1224 | 1143 | 380 | 99 | 97 |
|  |  | BuPAG 7 variant 18 | 1227 | 1032 | 343 | 81 | 87 |
|  |  | BuPAG 7 variant 19 | 1227 | 1143 | 380 | 100 (ADO67796.1) | 96 (AAI33470.1) |
|  |  | BuPAG 7 variant 20 | 1227 | 1143 | 380 | 100 | 96 |
|  |  | BuPAG 7 variant 21 | 1117 | 1104 | 367 | 81 | 87 |
|  |  | BuPAG 7 variant 22 | 1227 | 1143 | 380 | 95 | 92 |
|  |  | BuPAG 7 variant 23 | 1227 | 1143 | 380 | 99 | 97 |
|  |  | BuPAG 7 variant 24 | 1224 | 1140 | 379 | 97 | 94 |
|  |  | BuPAG 7 variant 25 | 1227 | 1143 | 380 | 100 | 96 |
|  |  | BuPAG 7 variant 26 | 1228 | 1002 | 333 | 99 | 95 |
|  |  | BuPAG 7 variant 27 | 1227 | 1143 | 380 | 98 | 94 |
|  |  | BuPAG 7 variant 28 | 1227 | 1143 | 380 | 94 | 91 |
|  |  | BuPAG 7 variant 29 | 1227 | 1143 | 380 | 99 | 96 |
| 6 | BuPAG 8 | BuPAG 8 variant 1 | 1227 | 1143 | 380 | 88 | 89 |
|  |  | BuPAG 8 variant 2 | 1227 | 1143 | 380 | 90 | 87 |
|  |  | BuPAG 8 variant 3 | 1227 | 1143 | 380 | 99 (ADO67797.1) | 93 (NP_788803.1) |
|  |  | BuPAG 8 variant 4 | 1227 | 1143 | 380 | 95 | 90 |
|  |  | BuPAG 8 variant 5 | 1227 | 1143 | 380 | 99 | 92 |
| 7 | BuPAG 9 |  | 1227 | 1143 | 380 | 99 (XP_006050181.1) | 94  (NP_788793.1) |
| 8 | BuPAG13 |  | 1227 | 1143 | 380 | 99  (ADO67802.1) | 86  (XP_002699292.1) |
| 9 | BuPAG15 |  | 1127 | 1143 | 380 | 99  (ADO67804.1) | 94  (DAA13837.1) |
| 10 | BuPAG16 | BuPAG 16 variant 1 | 1227 | 1143 | 380 | 87 | 95 |
|  |  | BuPAG 16 variant 2 | 1227 | 1143 | 380 | 87 | 95 |
|  |  | BuPAG 16 variant 3 | 1227 | 1143 | 380 | 87 (ADO67805.1) | 96 (DAA13834.1) |
|  |  | BuPAG 16 variant 4 | 1227 | 1143 | 380 | 87 | 92 |
|  |  | BuPAG 16 variant 5 | 1227 | 1143 | 380 | 88 | 94 |
|  |  | BuPAG 16 variant 6 | 1227 | 1143 | 380 | 89 | 93 |
| 11 | BuPAG 18 | BuPAG 18 variant 1 | 1224 | 1140 | 379 | 99 | 97 |
|  |  | BuPAG 18 variant 2 | 1203 | 1119 | 372 | 97 | 95 |
|  |  | BuPAG 18 variant 3 | 1224 | 1140 | 379 | 89 | 91 |
|  |  | BuPAG 18 variant 4 | 1224 | 1140 | 379 | 100 (ADO67807.1) | 97 (NP_001077166.1) |
|  |  | BuPAG 18 variant 5 | 1227 | 1143 | 380 | 92 | 91 |
|  |  | BuPAG 18 variant 6 | 1227 | 1143 | 380 | 92 | 90 |
|  |  | BuPAG 18 variant 7 | 1228 | 1104 | 367 | 91 | 91 |
|  |  | BuPAG 18 variant 8 | 1203 | 1119 | 372 | 97 | 96 |
|  |  | BuPAG 18 variant 9 | 1224 | 1140 | 379 | 99 | 97 |
|  |  | BuPAG 18 variant 10 | 1227 | 1143 | 380 | 92 | 90 |
|  |  | BuPAG 18 variant 11 | 1224 | 1140 | 379 | 99 | 97 |
| 12 | Novel BuPAG |  | 1224 | 1140 | 379 | 79  (XP_006076403.1) | 78  (DAA13834.1) |
