# Supplementary material for: Primary structures of different isoforms of buffalo pregnancy-associated glycoproteins (BuPAGs) during early pregnancy and elucidation of the 3-dimensional structure of the most abundant isoform BuPAG 7
Source: PLoS One. 2018 Nov 7;13(11):e0206143. doi: 10.1371/journal.pone.0206143 (PMC6221303; doi:10.1371/journal.pone.0206143)
Supplement: S1 Fig — Total RNA and amplified cDNA representative of three stages of pregnancy i.e. 45 days, 75 days and 90 days: A. Total RNA isolated from cotyledonary tissue at 45 days (lane 1), 75 days (lane 2) and 90 days (lane 3) separated on 1.2% agarose gel representing 28S and 18S intact rRNA bands. B. Agarose gel electrophoresis of PCR amplified BuPAG genes. PCR product of size ~1.2 kb was observed at 45 days (lane 1), 75 days (lane 2) and 90 days (lane 3) pregnancy. Lane 4 represents the DNA ladder. (DOCX) [file pone.0206143.s004.docx]

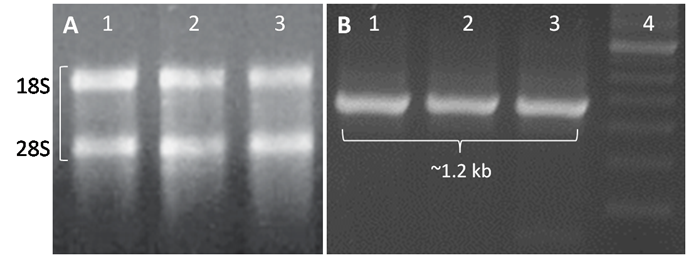


**S1 Fig: Total RNA and amplified cDNA representative of three stages of pregnancy i.e. 45 days, 75 days and 90 days:** **A.** Total RNA isolated from cotyledonary tissue at 45 days (lane 1), 75 days (lane 2) and 90 days (lane 3) separated on 1.2% agarose gel representing 28S and 18S intact rRNA bands. **B.** Agarose gel electrophoresis of PCR amplified BuPAG genes. PCR product of size ~1.2 kb was observed at 45 days (lane 1), 75 days (lane 2) and 90 days (lane 3) pregnancy. Lane 4 represents the DNA ladder.
