# Supplementary material for: Primary structures of different isoforms of buffalo pregnancy-associated glycoproteins (BuPAGs) during early pregnancy and elucidation of the 3-dimensional structure of the most abundant isoform BuPAG 7
Source: PLoS One. 2018 Nov 7;13(11):e0206143. doi: 10.1371/journal.pone.0206143 (PMC6221303; doi:10.1371/journal.pone.0206143)
Supplement: S2 Fig — Percent identity matrices of variants belonging to (A) BuPAG 2 (B) BuPAG 7 (C) BuPAG 8 (D) BuPAG 16 and (E) BuPAG 18. (DOCX) [file pone.0206143.s005.docx]

**(A)**

**
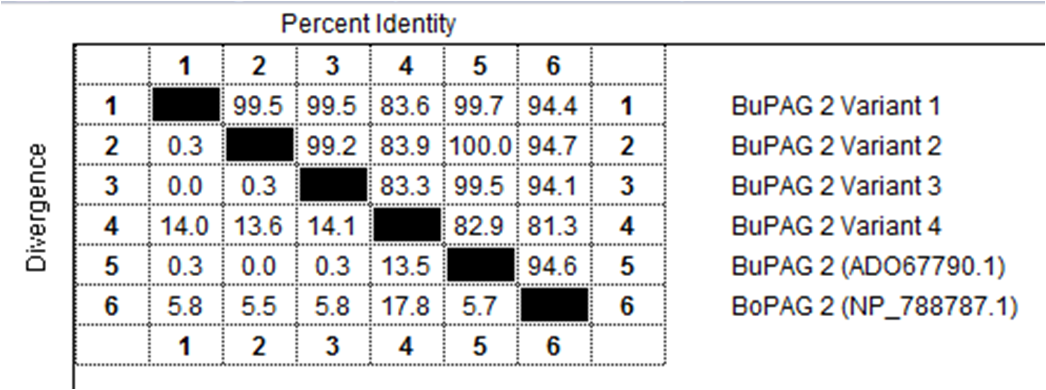
**

**(B)**

**
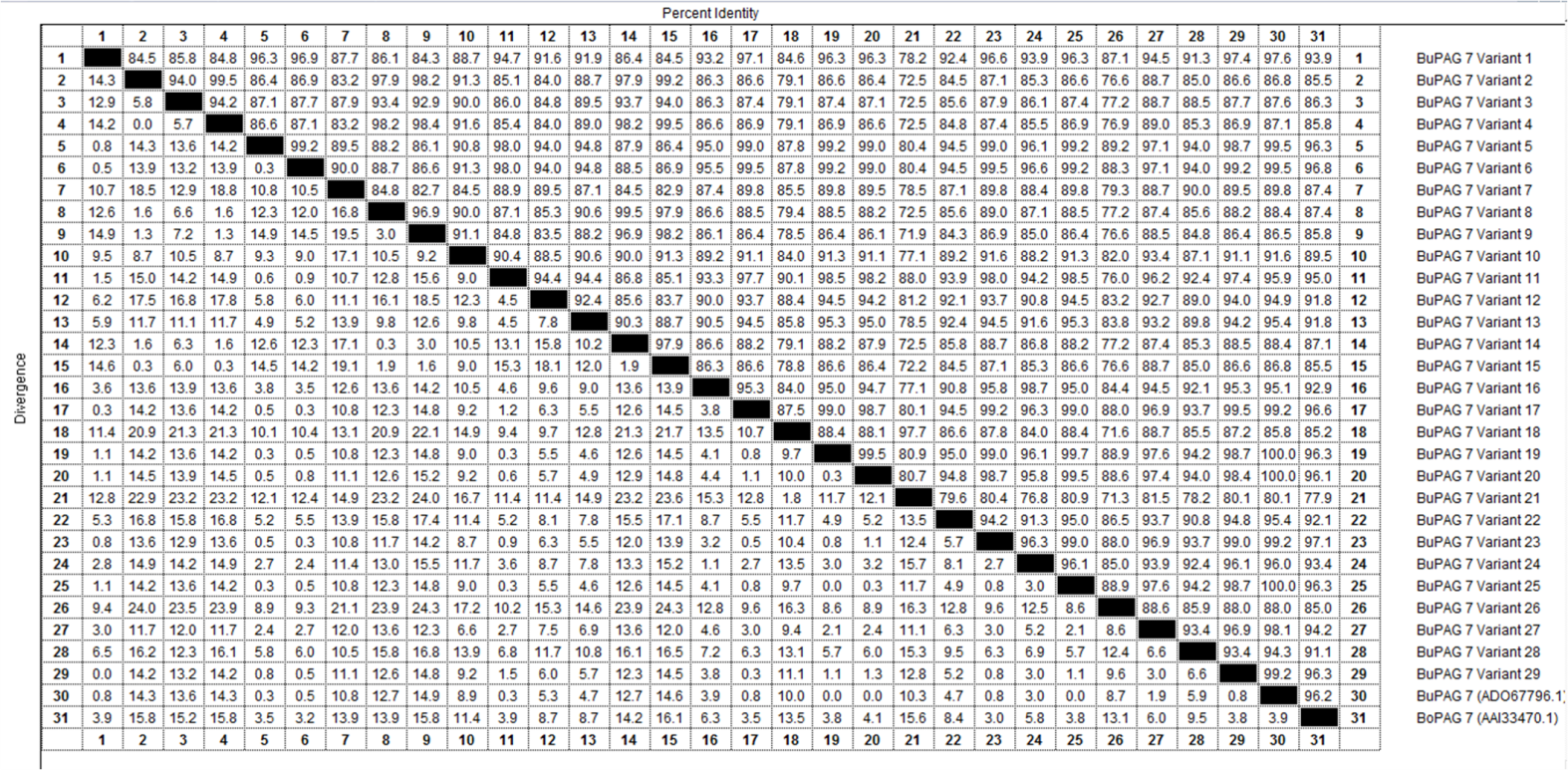
**

**(C)**

**
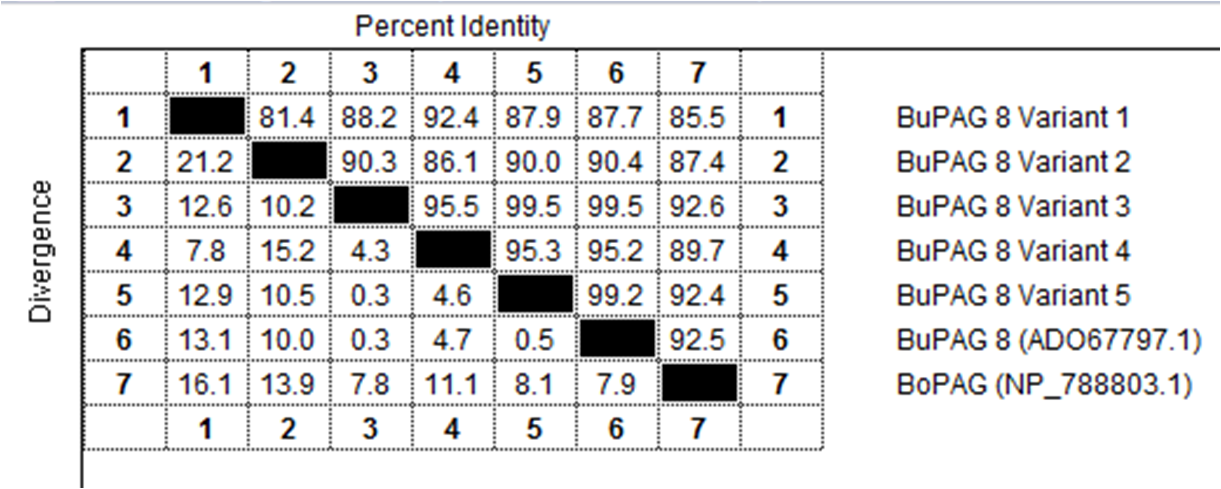
**

**(D)**

**
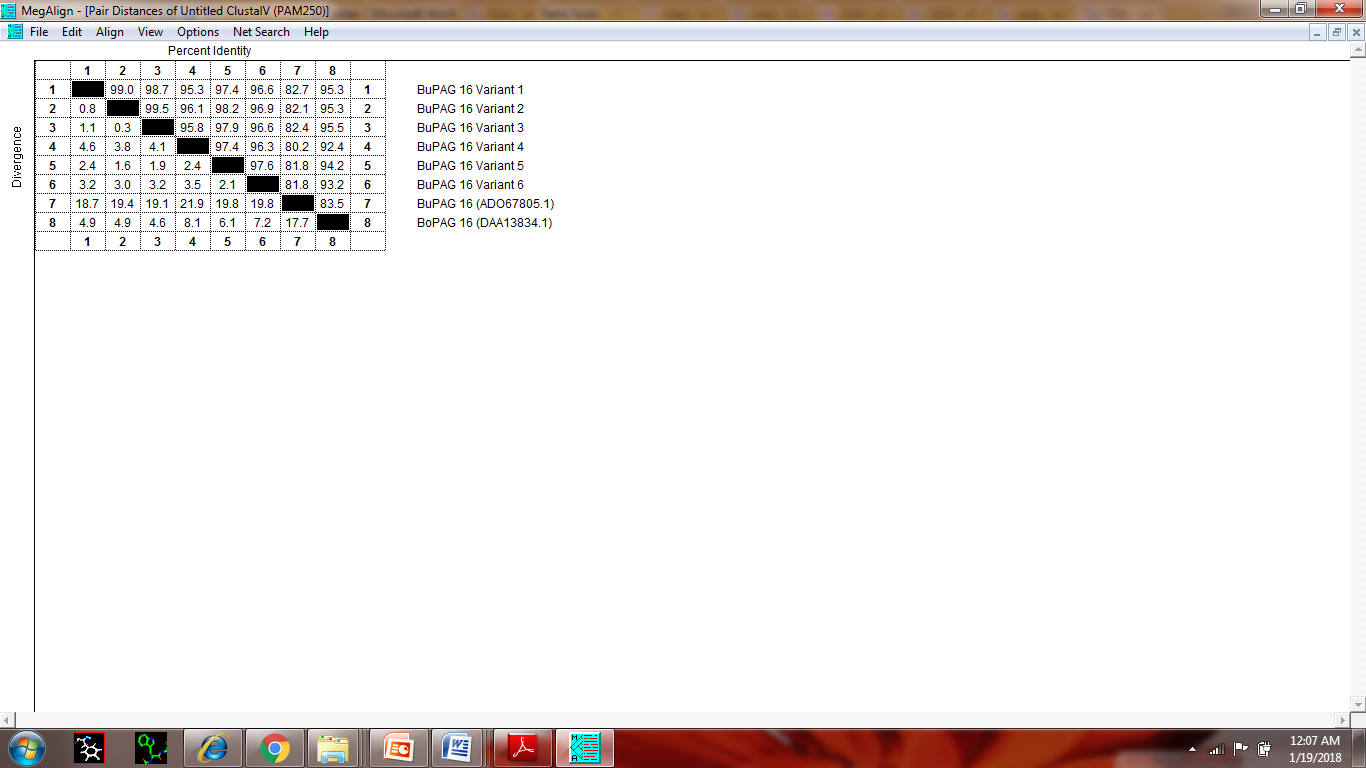
**

**(E)**

**
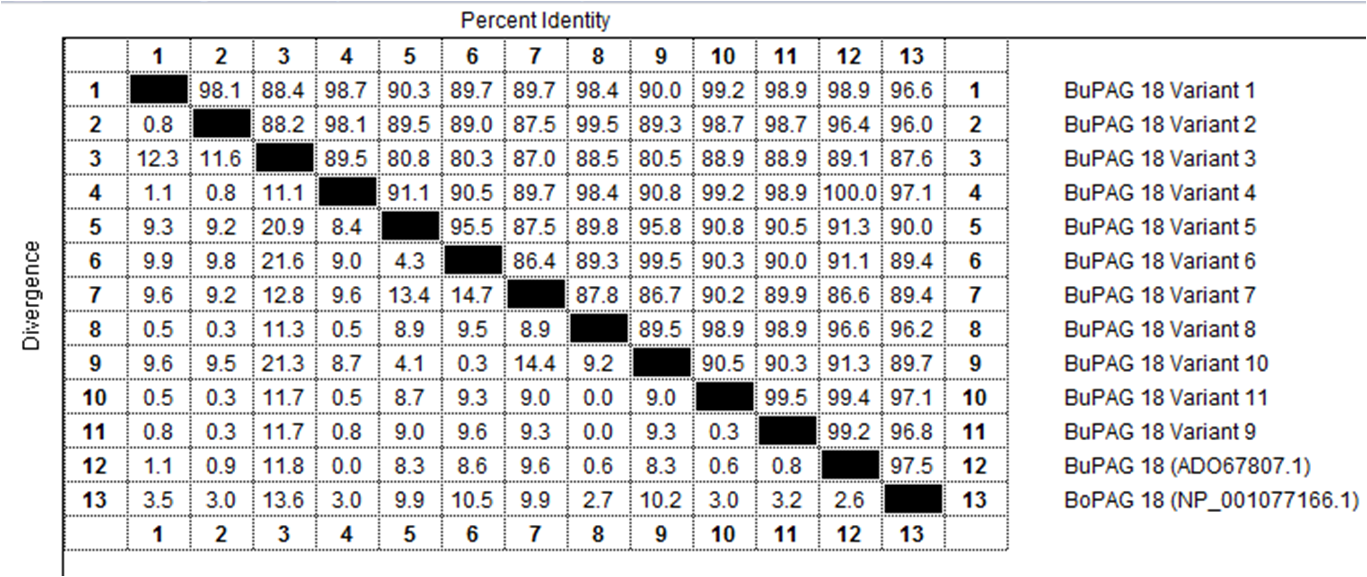
**

**S2 Fig: Percent identity matrices of variants belonging to (A)** BuPAG 2 **(B)** BuPAG 7 **(C)** BuPAG 8 **(D)** BuPAG 16 and **(E)** BuPAG 18
