# Supplementary material for: Primary structures of different isoforms of buffalo pregnancy-associated glycoproteins (BuPAGs) during early pregnancy and elucidation of the 3-dimensional structure of the most abundant isoform BuPAG 7
Source: PLoS One. 2018 Nov 7;13(11):e0206143. doi: 10.1371/journal.pone.0206143 (PMC6221303; doi:10.1371/journal.pone.0206143)
Supplement: S3 Fig — The tree was drawn to scale, and the numbers on the branches represent the confidence levels obtained from the bootstrap analysis (1000 replicates). (DOCX) [file pone.0206143.s006.docx]

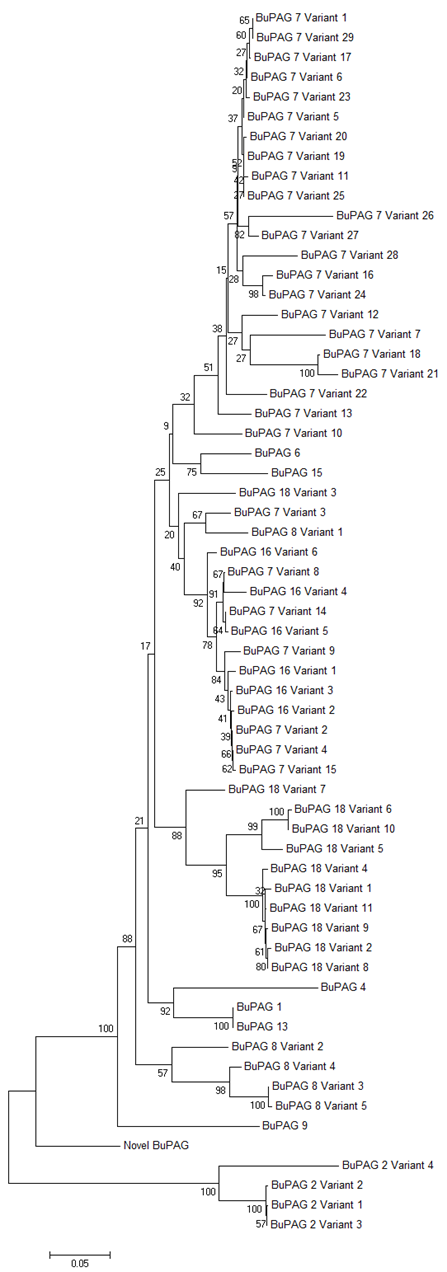


**S3 Fig:** **Evolutionary relationships among different isoforms of BuPAGs and their variants:** The tree was created from the deduced amino acid sequences by the Neighbor Joining method in the MEGA 4.0 program. The tree was drawn to scale, and the numbers on the branches represent the confidence levels obtained from the bootstrap analysis (1000 replicates).
